# Ten recommendations for organising bioimaging data for archival

**DOI:** 10.12688/f1000research.129720.1

**Published:** 2023-10-23

**Authors:** Paul K. Korir, Andrii Iudin, Sriram Somasundharam, Simone Weyand, Osman Salih, Matthew Hartley, Ugis Sarkans, Ardan Patwardhan, Gerard J. Kleywegt

**Affiliations:** 1 EMBL-EBI, Wellcome Genome Campus, Hinxton, Cambridgeshire, CB10 1SD, UK

**Keywords:** Organising data, public archiving, data deposition, open data, bioimaging, EMPIAR, BioImage Archive, BioStudies

## Abstract

Organised data is easy to use but the growth of bioimaging, with improvements in instrumentation, detectors, software and experimental techniques has resulted in an explosion in the volumes of data being generated, making this an elusive goal. This guide offers a handful of recommendations whose implementation would contribute towards better organised data in preparation for archival. Based on our experience archiving large image datasets in EMPIAR, the BioImage Archive and BioStudies, we propose a number of strategies that we believe would make future data depositions more useful to the bioimaging community and that may also find use in other data-intensive disciplines. To facilitate the process of analysing data organisation, we present bandbox, a Python package that provides users with an assessment of their data by flagging potential issues that could be addressed before archival.

## Introduction

Resources such as EMPIAR ( Iudin *et al.*, 2016; Iudin *et al.*, 2023) and the BioImage Archive ( Ellenberg *et al.*, 2018; Hartley *et al.*, 2022) provide a valuable service to the life-science community by supporting the archival and reuse of imaging data, often acquired at considerable cost, in line with the aspirations of the FAIR Guiding Principles ( Wilkinson *et al.*, 2016). There are numerous advantages and benefits to reusing bioimaging data, including more economical use of limited resources such as instrumentation and highly skilled technical staff. Moreover, specimens may be unique, costly to acquire, or difficult to reproduce, meaning that such data may only be accessible via archives. Archived data can be mined for reanalysis, verification and validation, and for development of new analytical techniques and software tools, such as machine learning model training. Reuse of such data may also lead to improvements in how it is produced, both technologically and methodologically. As practitioners in bioimaging data archiving, it is our experience that handling large datasets presents several data-management challenges, particularly in recent years with the rapidly increasing volumes of bioimaging data ( Ellenberg *et al.*, 2018). For example, it took eight years for EMPIAR to archive a total of one petabyte of data, but the second petabyte took only 14 months ( Iudin *et al.*, 2023). Bioimaging datasets may comprise numerous and sometimes very large files in a variety of, sometimes proprietary, formats. Individual files may include multiple channels and time points and data and metadata from several specimens. Besides the raw image data, there may also be a need to archive processed data, reconstructed 3D volumes, segmentations, particle stacks and other derived or related data.

There are two related but distinct avenues for organising data: labelling (metadata) and arranging data items (order). Metadata are essential to make the data useful even though metadata standards are difficult to enforce. Therefore, metadata standardisation has received a lot of attention with initiatives such as Bioschemas, an effort to improve findability of datasets via standardised textual annotations, and MIAME ( Brazma *et al.*, 2001), recommendations for minimal metadata describing a microarray experiment, and the overarching FAIR Guiding Principles ( Wilkinson *et al.*, 2016). For bioimaging, REMBI ( Sarkans *et al.*, 2021) provides community-supported recommendations on how to describe all aspects of bioimaging experiments including sample preparation, data processing and analysis. Whereas there are several ongoing efforts towards standardising bioimaging data formats (OME-NGFF ( Moore *et al.*, 2021), DVID ( Katz & Plaza, 2019), HDF5 ( Pietzsch *et al.*, 2015), etc.), we know of no efforts towards harmonising how to organise datasets for maximum usefulness with archival in mind. The organisation (order) of data is usually taken for granted and it falls upon refinements of the metadata to bear the burden of meaningfully describing the data.

## Motivation

Good organisation (order) of data improves its usefulness and is the responsibility of the data depositors. Depositors are best placed to present data in a way that adequately captures the experimental design and outcomes. Organising a dataset to minimally convey a structure in line with the actual experimental output can improve its usability while the bulk of meaningful attributes can be expressed in the metadata. The degree of usefulness depends directly on the quality of organisation, and thoughtful consideration of users’ needs improves that usefulness. Good organisation also gives a dataset transparency and understandability: users are able to immediately distinguish the various experimental categories as well as plan how to analyse the data. Therefore, it helps to have a clear perspective of the various classes of users.

In general, we consider three classes of users: *intra-domain scientists*, *inter-domain scientists* and *extra-domain scientists* ( Datta *et al.*, 2021). (For the purpose of this article, we will refer to any such user of a dataset as a ‘scientist’, interested in extracting some knowledge from the archived data.) Intra-domain scientists are familiar with key attributes of the data and may be able to quickly assess the usefulness of a dataset. An example would be a structural biologist mining an electron cryo-tomogram to extract sub-volumes that have not been previously studied. Inter-domain scientists may want to mine the data for purposes tangential to some other domain. For example, a genomicist may want to include structural analyses in a genomics study and may turn to raw imaging data to accomplish this. Extra-domain scientists are only interested in data for its technical properties, i.e., for some purpose completely unrelated to the original purpose of the data’s collection. A computer scientist, for example, may want to assess the performance of a learning algorithm on fluorescent microscopy images when performing some classification task. It is likely to be a challenge to optimise the organisation of data for all classes of users simultaneously. In practice, organising the data to be useful to scientists with the least familiarity with the domain will most likely advance its usefulness for all classes of scientists and can thus be a good aspiration.

The task of organising data consists of making trade-offs in the use of ‘ways and means’ of effecting the organisation. We will refer to these ‘ways and means’ as *organisational resources.* A simple example would be the use of alphabetic ordering when organising a set of strings; the natural ordering according to some alphabet is our organisational resource and we exploit the fact that most users will perceive this as a convenience when traversing the data. In less trivial organisational tasks, we need to express complex relationships between the entities to be organised. For instance, a dataset that consists of the experimental measurements resulting from a sequence of treatments on a set of specimens measured at various points in time requires the use of specimen, treatment and time point identifiers as well as other experimental aspects (data formats, alternative perspectives, transformations of the data such as changes in units, etc.) to be captured in such a way as to preserve the main experimental relationships. In that case, we can expand our set of organisational resources to include a folder hierarchy and file formats in addition to the set of symbols (letters, numerals, punctuation, literal symbols, uppercase and lowercase and so on) used to create the various identifiers. Ideally, we would like to keep repetition to a minimum so that the nature of the experiment can be readily discerned.

The manner in which organisational resources are used affects the usability of the resulting organisation: using too few of them will obscure the meaning of the organisation while using too many will overwhelm potential users. For example, including redundant folders along any part of the hierarchy (folders that contain only a single folder which in turn contains the actual data) makes it tedious to navigate through a dataset. On the other hand, dumping all files into one folder will make it difficult for the end user to distinguish between groups of semantically related files, especially when thousands of files are present. Similarly, naming files and folders by referring to entities inaccessible to its intended users (e.g., private machine names or private accession codes that external users will not have access to or even fathom) consumes precious ‘name space’ without conveying any useful information. Organising data is thus an investment of time and effort with the ultimate aim of improving the usefulness of the data.

We can therefore formulate the organisation task as follows: *given a set of related data items associated with an experiment, how may they be organised to best convey their relationships using as few organisational resources as possible while maximising their usability?*

To achieve this, we define the term *facet* to refer to the various attributes germane to the experiment which may be included in the folder and file names. A non-exhaustive list of facets are: specimen names ( *organism, tissue, cell type/line*), experimental roles ( *treatments vs. controls*), time ( *developmental status, date, elapsed time*), processing status ( *raw data, by algorithm, procedure*), generally available equipment ( *microscopes, detectors, preparation equipment model names*), replicates, file types ( *3D volumes, particle stacks*), names of software used for processing, and so on.

This guide attempts to solve the organisation task by providing 10 recommendations that arise from our experience of handling hundreds of large image datasets in the public archives EMPIAR, BioImage Archive and BioStudies ( Sarkans *et al.*, 2018). Ideally, we would like to organise potentially numerous and voluminous data to maximise ease of use so as to facilitate the user’s ability to:

1. quickly identify the suitability of (subsets of) the data;
2. clearly distinguish between the various facets of the data;
3. quickly verify the usefulness of the data (e.g., thumbnails, previews, summaries, READMEs, LICENCE files);
4. retrieve only relevant subsets of the data.

This guide does not offer any recommendations for a detailed schema to describe experimental and analytical procedures; those may be captured in metadata for the various archives. Neither does it describe how to decide which experimental facets are appropriate (these are part of the experimental design), nor does it attempt to describe how to achieve organisation for automated analysis (we assume that the resulting organisation will be consumed by humans). It also ignores the universe of image formats in use and mainly includes examples from our experience archiving bioimaging data, but we anticipate it may be useful across other imaging disciplines. Good organisation improves data structure and format predictability and may facilitate automated processing. Therefore, our guide is intended to lead towards best practices rather than serve as a framework. Finally, this guide does not aim to achieve standardisation. We believe it is more practical to have a set of best practices and leave it up to the data authors to decide how best to apply them.

We believe that the recommendations outlined here may be of value to two principal groups of users: 1) data depositors, who need to design and prepare their data to improve its usability to the community, and 2) technologists (hardware, software and methods developers), who, by considering these recommendations in their designs, can greatly facilitate data organisation at the source.

**Figure 1.**
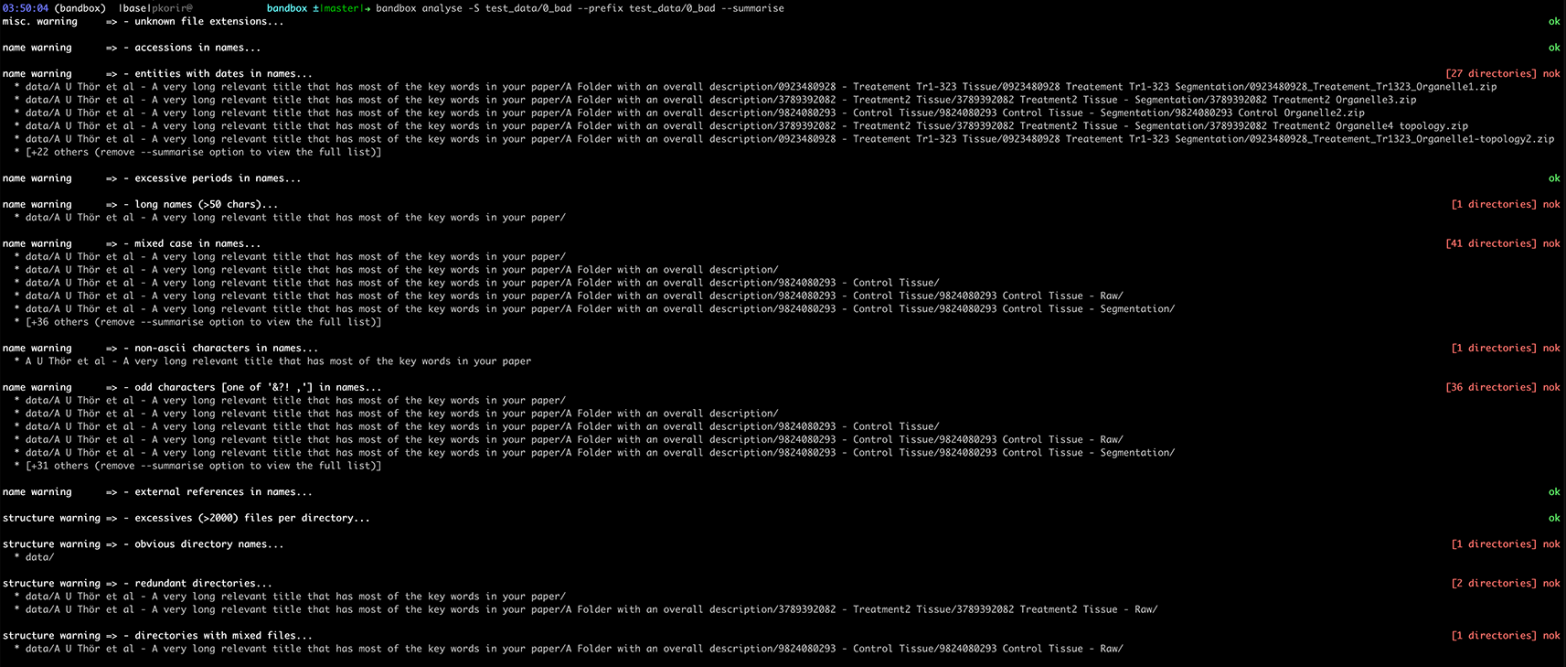
Example of a dataset with organisational red flags identified by bandbox such as use of spaces or non-ASCII characters, redundant directories and so on with an indication of the number of such entities found. The actual example dataset is provided with the bandbox source code (ASCII – American Standard Code for Information Interchange).

**Figure 2.**
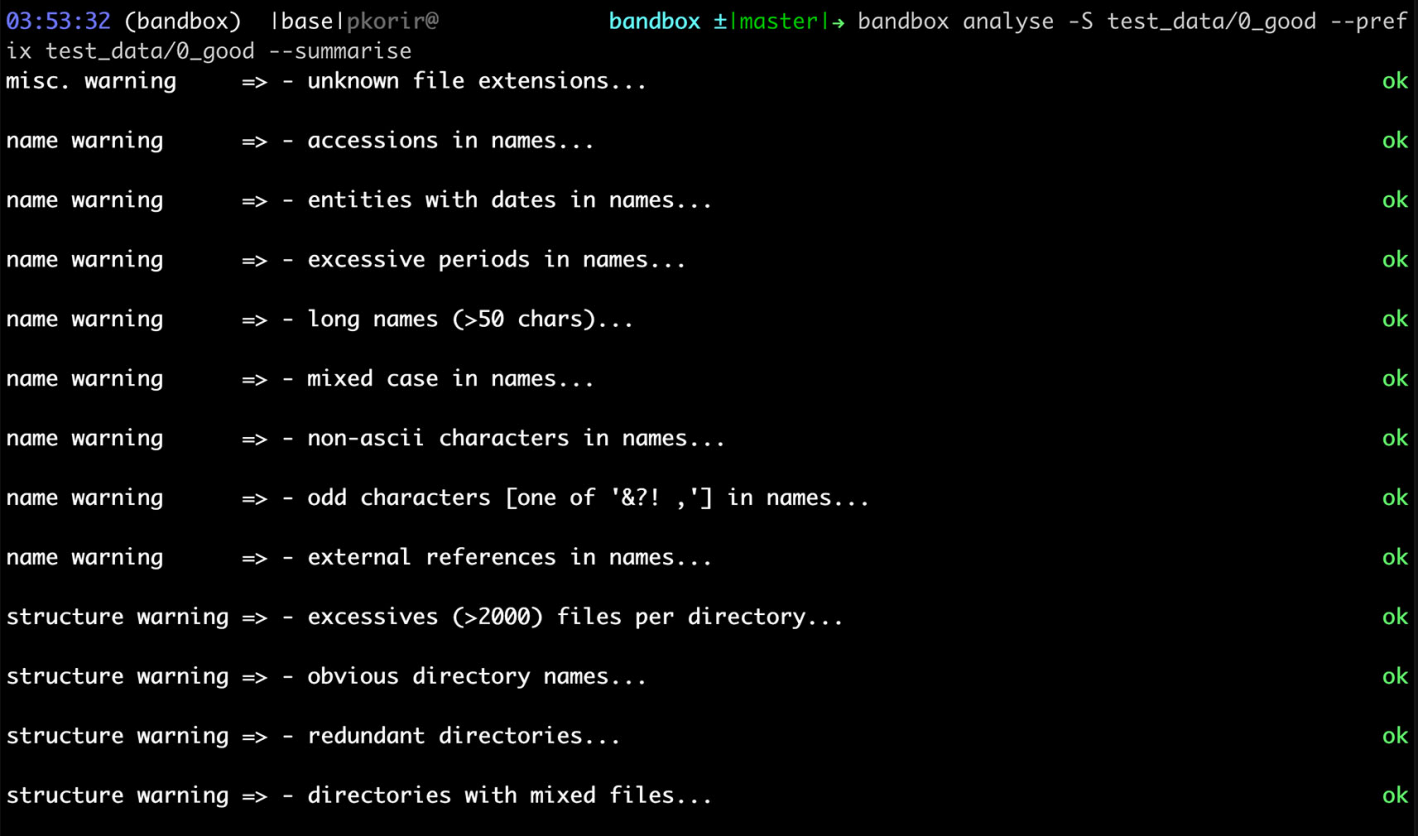
Example of a dataset with no red flags as inferred by bandbox . The actual example dataset is provided with the bandbox source code.

To make our recommendations practical, we have developed bandbox ( Korir *et al.*, 2022), an open-source command-line interface (CLI) tool to help users understand how they can improve the organisation of their data in preparation for archival. The program offers two CLI commands: view and analyse . The view command displays a tree of a directory and all its contents; for every non-empty directory with files, bandbox provides a summary of the number of files in it, including a list of all the file formats encountered. The analyse command provides a listing of possible issues grouped into categories in line with those specified in the Recommendations section of this article. bandbox examines the tree associated with the nested hierarchy of files and folders in a dataset and then concurrently runs various heuristics on the tree which are controlled by configurations that the user may modify. The results produced by the analyse command are only suggestions for improvement; we understand that there may be practical limitations to implementing some of the suggested improvements as well as good reasons for keeping the data as is. Figures 1 and 2 show screenshots of the results of running bandbox on two different datasets.

## Recommendations

We will motivate our guide by referring to a fictitious EMPIAR dataset. This dataset has a clear structure, but we propose that it can be further improved following the recommendations in the guide below.

**Figure 3.**
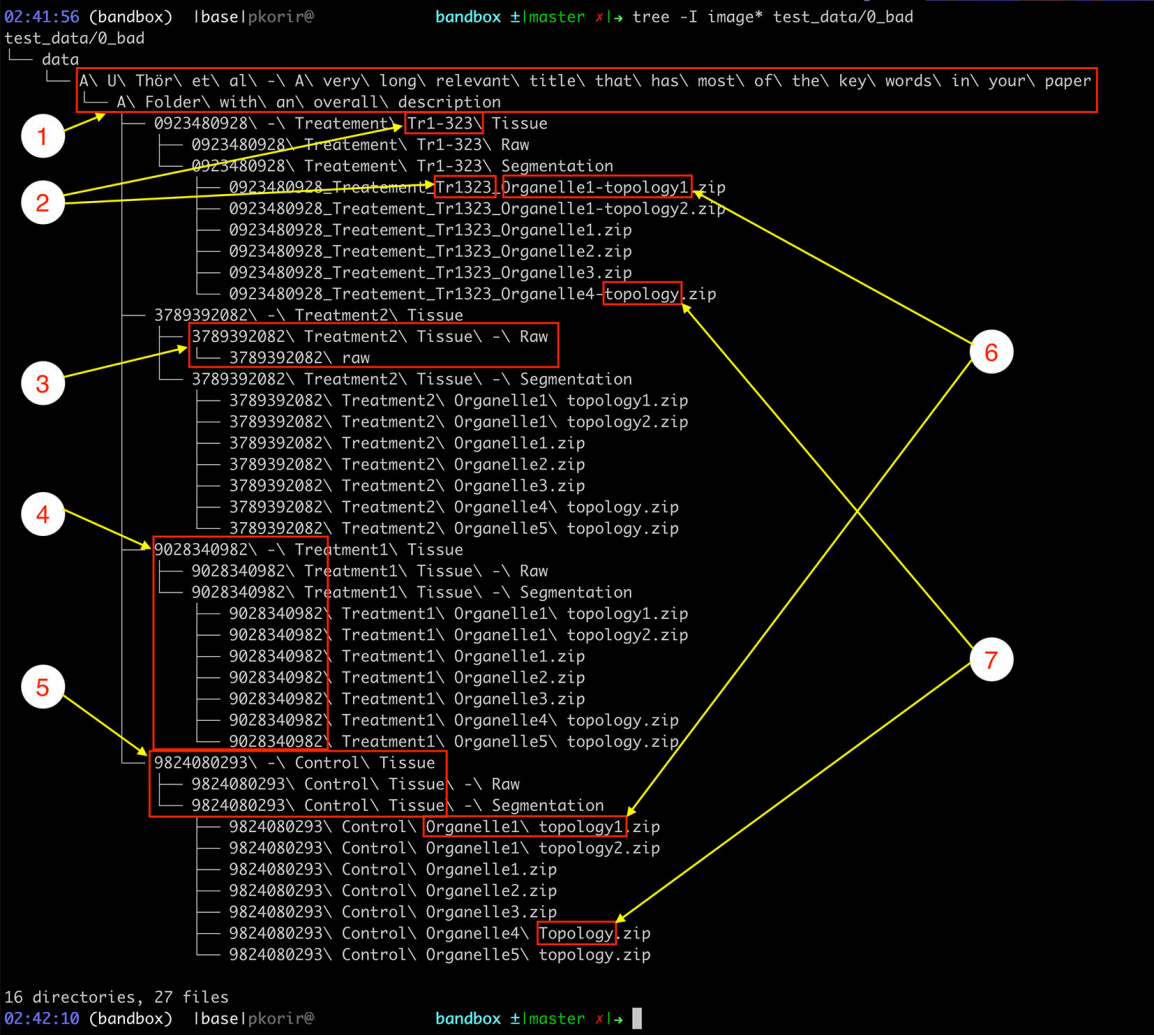
Illustration of some of the ways in which subtle features of data organisation impact its usability. Issues include: 1) long file/folder names with spaces, non-ASCII characters (ö) and redundant directories (ASCII – American Standard Code for Information Interchange); 2) obscure sequences with inconsistent spelling, 3) inconsistency in folder hierarchy, 4) obscurity through meaningless symbol sequences, 5) verbosity in names, 6) subtle differences in spelling (in this case, a hyphen) and 7) inconsistency in typography due to character case and inclusion of different separator characters, e.g., spaces vs hyphens. See text for more details.

Our goal is to improve the file/folder structure shown in Figure 3 to better convey the relationships between the experimental facets while economising the organisational resources available. For clarity, we have refrained from listing several thousand raw TIFF files in the folders designated ‘Raw’ .

The example dataset illustrates several properties of its organisation that undermine the goal of being usable:

• **Verbosity** typically presented by repetition of references which may be resolved using the file hierarchy, such as:
  ○ Folders containing only a single folder which in turn contains the folder with the actual data. The child folder of ‘data’ only has the folder ‘A U Thör et al …’ in it that contains the folder ‘A folder with an overall description’ which has the actual data.
  ○ Very long names of files/folders. The full path of the file ‘data/A U Thör et al - A very long relevant title that has most of the keywords in your paper/A Folder with an overall description/0923480928 - Treatement Tr1-323 Tissue/0923480928_Treatement_Tr1323_Organelle1-topology1.zip’ is ‘0923480928 Treatement Tr1-323 Segmentation/0923480928_Treatement_Tr1323_Organelle1-topology1.zip’, which might be outside the limits of legacy software; e.g., IMOD ( Mastronarde, 2006) has a limit of 320 characters for input file names.
  ○ Repetition of identifiers along the path. In the previous example, half of the files repeat the identifier ‘0923480928’ that conveys no meaningful information and which, if required at all, should only appear in the appropriate parent folder name.
• **Ambiguity** occurs through incomplete identifiers either due to typos or non-standard characters.
  ○ Is ‘Tr1-323’ the same as ‘Tr1323’ ?
  ○ Use of spaces and non-ASCII characters can make processing the data complicated because of how software may handle path names with spaces. ASCII stands for the American Standard Code for Information Interchange and consists of plain characters used in many languages.
• **Inconsistency** is perhaps the most common issue and is usually the result of manually introduced errors such as changes in spelling, e.g., naming similar folders ‘tomo’ and ‘tomogram’ for related files. In the above example we have:
  ○
‘Topology’ and ‘topology’

  ○
‘Treatment’ vs ‘Treatement’

  ○
‘Tr1-323’ and ‘Tr1323’

  ○ Inconsistency may also be observed in folder structure. For example, only one of the treatment folders (the one with ‘3738932082’ in the name) has an extra child folder, breaking the trend of the others.
• **Obscurity** tends to occur through the use of identifiers with no obvious meaning, e.g., references to external resources such as figure numbers in a related paper, machine identifiers, script names, etc.
  ○ The numerical identifiers such as ‘0923480928’ have no obvious meaning in the context of the dataset.
  ○
‘Tr1-323’ may be an external reference but its meaning is unclear.
  Understandably, in certain cases such obscurity may be useful to keep identifiers which convey additional information. For example, in electron cryo-microscopy (cryo-EM) pipelines, the dataset may consist of multiple subsets obtained with different open-source software, e.g. particle picking by EMAN2 ( Tang *et al.*, 2007), beam-induced motion-correction by MotionCorr ( Li *et al.*, 2013), contrast-transfer function (CTF) correction by gCTF ( Zhang, 2016), classification by RELION ( Scheres, 2012), reconstruction by cryoSPARC ( Punjani *et al.*, 2017), etc.

The 10 recommendations we present below are divided into four groups: *planning* (one recommendation), *structure* (three recommendations), *naming* (three recommendations) and *miscellaneous* (three recommendations). We have provided further guidance within each group for related concepts.

### Planning

**(1) Design before data collection.** Plan beforehand, if possible, how the data may be structured.

a. If the experimental facets are known prior to data collection, the organisation suggestions that follow below will be easier to apply once and for all; it is harder to reorganise data, especially voluminous data on multiple networked drives or in a cloud resource after collection. At a minimum, consider organising the few top-level directories in terms of the experimental facets prior to archival.
b. Consider employing a naming convention within a research group to ensure that data is consistent between creators of the data. This can even be specified in the microscope’s software to include imaging parameters in the file names automatically such as a base name, date and/or time, imaging parameters (e.g., resolution, section size) or even free text, among many others. We invite software vendors/creators who have not already done so to consider incorporating organisational concerns into their software that take these recommendations into account.

### Structure

This section contains recommendations to address the hierarchical organisation of files and folders only.

**(2) Containing folder.** Consider having one parent folder into which all sub-datasets are located. Such a container folder is also a good location to include auxiliary data that apply to the collection such as README or integrity (see recommendation 10) files, which provide users with the context of the data organisation.

**(3) Folder depth.**

a. Consider limiting the folder depth to a reasonable maximum. As a rule of thumb, three to four directory levels is adequate for most applications but the fewer the better.
b. Consider excluding folders which do not convey any additional information. For example, consider a dataset having only TIFF files. Including a folder called tiff in the path <condition>/tiff/files*.tif is redundant. By contrast, if the file format is instrumental then <condition>/<format1>/<files_of_format1> and <condition>/<format2>/<files_of_format2> and so on is meaningful.
c. Consider an upper limit on the number of files in a folder and if necessary split large directories so they do not contain more than a certain maximum number of files (e.g., 10,000). If, for instance, a folder has one million files then it may instead be organised as a folder ( parent_folder ) with 100 sub-folders ( child00 to child99 ), each containing 10,000 files. This is important because different file systems have different tolerances for handling large numbers of files. For example, the Second Extended Filesystem (ext2) imposes ‘soft’ limits of 10,000 files per directory because of the extra overhead when processing such large folders ( *The Second Extended Filesystem — The Linux Kernel Documentation* ). While modern file systems are capable of handling larger numbers of files, the re-usability of the data will increase when taking into account systems with more modest resources, such as web browsers that may need to list or process all files in a directory.

**(4) Folder contents.**

a. Consider grouping related files unless it is instrumental to keep them separated. For example, group files by specimen, filetype, experimental purpose (treatment, control), etc. It may be instrumental to separate different data types into different folders (e.g., one for micrographs and one for particle stacks). Further sub-folders may be necessary for single- and multi-frame micrographs, unaligned and aligned micrographs, etc.
b. Consider depositing data from different experimental techniques/sub-techniques as separate archive entries (e.g., single-particle data in one, tomography data in another). Most archives allow multiple separate entries to be linked or grouped.

### Naming

In this section, we provide some suggestions to improve the naming of files and folders.

**(5) Meaningful names.**

a. Consider naming files and folders using meaningful identifiers without specifying external references. For instance, while the name ‘Figure 5’ probably refers to a paper describing (some of) the data, users will require access to that manuscript, which may be behind a paywall. The names of files and folders should exclude any references that are tied to the instrument or your organisation, which are at best unhelpful for external users.
b. Consider avoiding ambiguous attributes such as dates and times particularly in folder names. Mass renaming of files with dates and times can become non-trivial particularly if such attributes have subtle variations for related files (e.g., as date/time stamps are incremented) and is therefore best avoided.

**(6) Naming symbols**.

a. Consider confining names to lowercase letters and numerals and replacing all spaces with underscores or hyphens for meaningful word (group) boundaries as this makes it substantially easier when working with the data. Preferably, consider underscores only for word boundaries and hyphens for keywords or other key attributes such as specimen names identifiable by the presence of a hyphen, e.g., covid-19 . Consistent use of case also improves readability ( Deissenboeck & Pizka, 2006).
b. Consider avoiding certain characters which could lead to unintended consequences during processing such as ampersands (&), spaces, exclamation marks (!) and question marks (?). In general, stick to printable alphanumeric ASCII characters and avoid non-ASCII characters (e.g., ü, å or non-Roman scripts).
c. Consider avoiding periods in names as this can lead to unpredictable behaviour for instance when attempting to determine formats. For example, while it is generally well known that the file file.tar.gz has two standard extensions, it may not be as widely known that file.ome.tiff, file.ome.tf2, file.ome.tf8 and file.ome.btf are all valid multi-extension bioimaging formats ( *OME-TIFF Specification — OME Data Model and File Formats 6.2.2 Documentation* ).
d. Consider an upper limit on the length of file and folder names. We propose a working upper limit of 50 characters. Even though modern operating systems have no limitations on the lengths of names, end users will still struggle typing very long names which increases the likelihood of transcription errors. In some cases, software that is widely used by the bioimaging community imposes limits on the number of characters for file paths, e.g., IMOD ( Mastronarde, 2006) imposes a file path limit of 320 characters. Bear in mind that, increasingly, users will interact with datasets via a web browser, which also has a practical limit (based on the device’s memory) on the number of files that can be selected in the browser’s select dialog.

**(7) Identity**.

a. Ensure consistency when naming files and folders so that similar folders at different depths have the same names.
b. Do not include personal identifiers in folder or file names.
c. Some words to consider for exclusion in the names of internal files/folders: ‘files’ , ‘data’ , ‘images’ etc. or other words that convey no additional meaningful information.
d. Think of folder names as applying to all the folders and files they contain as well: there should be no repetition in nested folder names, e.g., data/control.a/control.a.1/control.a.1.value/data/ ;
e. When providing 3D data as slices, consider zero-padding the slice identifiers which facilitates correct assembly. For example, consider an image with 1000 images at a resolution of MxN representing sections/slices of some volume; splitting this file should result in files of the form file0001.tif to file1000.tif . If zero-padding is missing or done incorrectly ( file1.tif to file1000.tif ), the order of slices will be lost on operating systems that apply lexicographic rather than numerical sorting. This can be fixed using the rename shell utility, e.g., rename file file00 file??.tif will convert all files with 01 to 99 to have 0001 to 0099 and so on. rename is available on most Linux distributions and may be installed on macOS using Homebrew or from the source code. On Windows systems the Bulk Rename Utility can be used.

### Miscellaneous

Finally, this section includes some tips on how to handle other aspects of organisation not covered in the previous sections.

**(8) Friendly file formats.**

a. Consider providing images in widely used file formats unless you are demonstrating a novel file format in which case it may be necessary to first get in touch with the archive to plan this. Additional information may be requested to provide users with guidelines on how to use and visualise the new format files including any conversion tools that are available and on providing the same data in a widely used file format as well.
b. Even for file types that are widely used, it may be helpful to stick to open formats to ensure that users without access to proprietary software will have access to the data.

**(9) Document your data.**

a. Consider including a README text file which provides an overview of how the data is organised.
b. Consider testing the usability of your data by asking a colleague to peruse your data to assess whether the organisation is clear. This can be achieved by asking the tester to describe their understanding of what the data presents.

**(10) Integrity.** If possible, consider including checksums, parity codes or hashes for each data file in a separate file, e.g., md5-sums.txt , imageset01.par2 or sha512-hashes.txt to facilitate content verification. These will allow users to verify that the data has not been corrupted during the deposition or download process. Each of these different ways to verify file integrity have corresponding tools available for all operating systems, but their operation is beyond the scope of this article ( Chi Lianhua & Zhu Xingquan, 2017).

**Figure 4.**
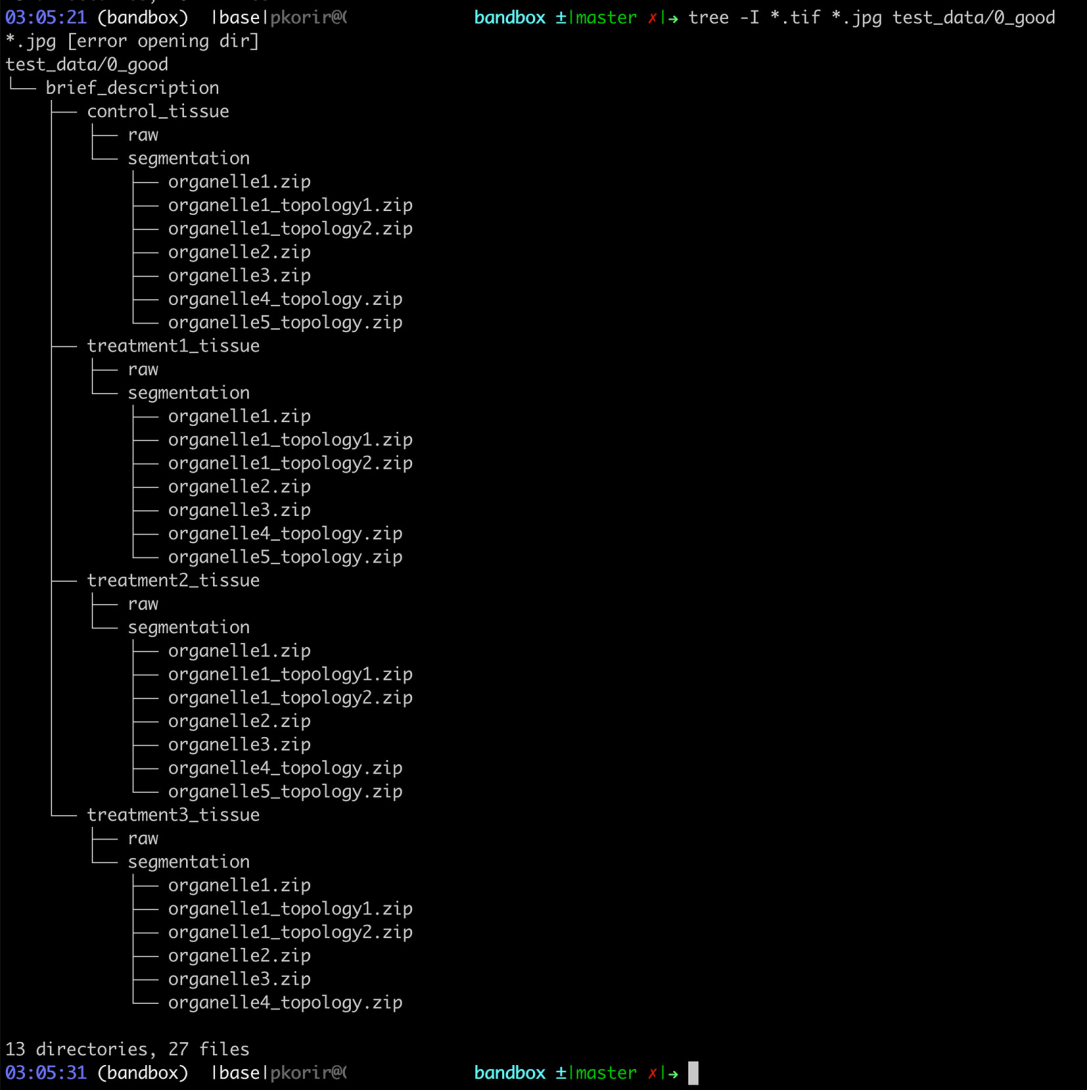
Tree representation of the data from Figure 3 reorganised by applying some of the 10 recommendations proposed.

Applying the recommendations above, we may revise the path:

data/A U Thör et al - A very long relevant title that has most of the keywords in your paper/A Folder with an overall description/0923480928 - Treatement Tr1-323 Tissue/0923480928_Treatement_Tr1323_Organelle1-topology1.zip’ is ‘0923480928 Treatement Tr1-323 Segmentation/0923480928_Treatement_Tr1323_Organelle1-topology1.zip

to:

data/brief_description/treatment3_tissue/segmentation/organelle1_topology1.zip ,

a reduction from 328 to 79 characters for the full path. The new organisation is presented in Figure 4.

## Conclusion

We hope that these 10 recommendations will only be the beginning of a broader discussion on how to organise bioimaging data in particular and experimental data in general for maximum usefulness, not just to the bioimaging community, but to the wider scientific community. Given the breadth of applications of bioimaging techniques, good organisation would go a long way to helping scientists from other disciplines to benefit from using bioimaging data. There is still considerable scope to develop better ways of not only organising data, but also representing it to enable automated data analysis.

## Data Availability

No data are associated with this article.

## Software availability

Software available from: https://pypi.org/project/bandbox

Source code available from: https://github.com/emdb-empiar/bandbox

Archived source code at time of publication: https://doi.org/10.5281/zenodo.7807541 ( Korir *et al.*, 2022).

License: Apache License 2.0
