# Ten recommendations for organising bioimaging data for archival

**DOI:** 10.12688/f1000research.129720.2

**Published:** 2024-02-27

**Authors:** Paul K. Korir, Andrii Iudin, Sriram Somasundharam, Simone Weyand, Osman Salih, Matthew Hartley, Ugis Sarkans, Ardan Patwardhan, Gerard J. Kleywegt

**Affiliations:** 1 EMBL-EBI, Wellcome Genome Campus, Hinxton, Cambridgeshire, CB10 1SD, UK

**Keywords:** Organising data, public archiving, data deposition, open data, bioimaging, EMPIAR, BioImage Archive, BioStudies

## Abstract

Organised data is easy to use but the rapid developments in the field of bioimaging, with improvements in instrumentation, detectors, software and experimental techniques, have resulted in an explosion of the volumes of data being generated, making well-organised data an elusive goal. This guide offers a handful of recommendations for bioimage depositors, analysts and microscope and software developers, whose implementation would contribute towards better organised data in preparation for archival. Based on our experience archiving large image datasets in EMPIAR, the BioImage Archive and BioStudies, we propose a number of strategies that we believe would improve the usability (clarity, orderliness, learnability, navigability, self-documentation, coherence and consistency of identifiers, accessibility, succinctness) of future data depositions more useful to the bioimaging community (data authors and analysts, researchers, clinicians, funders, collaborators, industry partners, hardware/software producers, journals, archive developers as well as interested but non-specialist users of bioimaging data). The recommendations that may also find use in other data-intensive disciplines. To facilitate the process of analysing data organisation, we present bandbox, a Python package that provides users with an assessment of their data by flagging potential issues, such as redundant directories or invalid characters in file or folder names, that should be addressed before archival. We offer these recommendations as a starting point and hope to engender more substantial conversations across and between the various data-rich communities.

## Introduction

Scientific data archival has a long history of providing publicly accessible storage of experimental data that typically involves manual and automated curation and annotation with appropriate metadata for reuse by others ( Whitlock *et al.* 2010; Rausher *et al.* 2010; Berman *et al.* 2014). In the area of bioimaging, global and public resources such as EMPIAR ( Iudin *et al.*, 2016, 2023) and the BioImage Archive ( Ellenberg *et al.*, 2018; Hartley *et al.*, 2022) provide a valuable service to the life-science community by supporting the archival and reuse of imaging data, often acquired at considerable cost, in line with the aspirations of the FAIR Guiding Principles ( Wilkinson *et al.*, 2016). There are numerous advantages and benefits to reusing bioimaging data, including more economical use of limited resources such as instrumentation and highly skilled technical staff. Moreover, specimens may be unique, costly to acquire, or difficult to reproduce, meaning that such data may only be accessible via archives. Archived data can be mined for reanalysis, verification and validation, and for development of new analytical techniques and software tools, such as machine learning model training. Reuse of such data may also lead to improvements in how it is produced, both technologically and methodologically. As practitioners in bioimaging data archiving, it is our experience that handling large datasets presents several data-management challenges, particularly in recent years with the rapidly increasing volumes of bioimaging data ( Ellenberg *et al.*, 2018). For example, it took eight years for EMPIAR to archive a total of one petabyte of data, but the second petabyte took only 14 months ( Iudin *et al.*, 2023). Bioimaging datasets may comprise numerous and sometimes very large files in a variety of, sometimes proprietary, formats. Individual files may include multiple channels and time points and data and metadata from several specimens. Besides the raw image data, there may also be a need to archive processed data, reconstructed 3D volumes, segmentations, particle stacks and other derived or related data.

There are two related but distinct avenues for organising data: labelling (metadata) and arranging data items (order). Metadata are essential to make the data useful even though metadata standards are difficult to enforce. Therefore, metadata standardisation has received a lot of attention with initiatives such as Bioschemas, an effort to improve findability of datasets via standardised textual annotations, MIAME ( Brazma *et al.*, 2001), recommendations for minimal metadata describing a microarray experiment, and the overarching FAIR Guiding Principles ( Wilkinson *et al.*, 2016). For bioimaging, REMBI ( Sarkans *et al.*, 2021) provides community-supported recommendations on how to describe all aspects of bioimaging experiments including sample preparation, data processing and analysis. Whereas there are several ongoing efforts towards standardising bioimaging data formats (OME-NGFF ( Moore *et al.*, 2021), DVID ( Katz & Plaza, 2019), BigDataViewer ( Pietzsch *et al.*, 2015), etc.), we know of no efforts towards harmonising how to organise datasets for maximum usefulness for archival in mind. The organisation (order) of data is usually taken for granted and it falls upon refinements of the metadata to bear the burden of meaningfully describing the data. Nevertheless, it is essential to maintain coherence between metadata and order of the data, for example in the naming of entities to facilitate meaningful navigation between the two.

## Motivation

Good organisation (order) of data improves its usefulness and is the responsibility of the data depositors. Depositors are best placed to present data in a way that adequately captures the experimental design and outcomes. For this reason, the recommendations that are outlined below are primarily targeted at data depositors as they are best positioned to structure the data meaningfully. Additionally, these recommendations are also aimed at developers of software tools that either produce the raw data (acquisition software on microscopes), process and transform it into a useful form, or extract meaningful domain insights (bioimage analysis). Organising a dataset to minimally convey a structure in line with the actual experimental output can improve its usability while the bulk of meaningful attributes can be expressed in the metadata. The degree of usefulness depends directly on the quality of organisation, and thoughtful consideration of the needs of users (i.e., those who consume the archived data) improves that usefulness. Good organisation also gives a dataset transparency and understandability: users can immediately distinguish the various experimental categories as well as plan how to analyse the data ( Petek *et al.*, 2022). Therefore, it helps to have a clear perspective of the various types of users.

In general, we consider three types of users: *intra-domain scientists*, *inter-domain scientists* and *extra-domain scientists* ( Datta *et al.*, 2021). (For the purpose of this article, we will refer to any such user of a dataset as a ‘scientist’, interested in extracting some knowledge from the archived data.) Intra-domain scientists are familiar with key attributes of the data and may be able to quickly assess the usefulness of a dataset. An example would be a structural biologist mining an electron cryo-tomogram to extract sub-volumes that have not been previously studied. Inter-domain scientists may want to mine the data for purposes relevant to some other domain. For instance, on the genomics side, using spatial transcriptomics imaging data for fine-grained localization of individual transcripts would be a possible scenario for an inter-domain scientist. Extra-domain scientists are only interested in data for its technical properties, i.e., for some purpose completely unrelated to the original purpose of the data’s collection. A computer scientist, for example, may want to assess the performance of a learning algorithm on fluorescent microscopy images when performing some classification task. It is likely to be a challenge to optimise the organisation of data for all types of users simultaneously. In practice, organising the data to be useful to scientists with the least familiarity with the domain will most likely advance its usefulness for all types of scientists and can thus be a good aspiration.

Organising data results in a hierarchical arrangement of data into files and folders. The visual properties of such an organisation influence the usability of the data. There are several considerations that affect the organisation:

• What are the sets of *symbols* used for naming the files and folders?
• What are the sets of relevant *named entities* these characters describe?
• How does the resulting *hierarchy*, defined using files and folders, capture the relationships between the named entities?

We can refer to the above as *organisational resources*, and it is through their judicious use that the data can become usable. Very long file names, potentially problematic characters, and deep nesting of folders are examples of how injudicious use of these resources can result in unusable data.

A simple example of how this is useful is the way most operating systems apply ordering of a directory’s contents either lexicographically or by other attributes such as date-time stamps. These take advantage of the familiarity that users have with the conventional ordering of these attributes. In non-trivial organisational tasks, we may need to express complex relationships between the entities at hand. For instance, a dataset that consists of the experimental measurements resulting from a sequence of treatments applied on a set of specimens measured at various points in time requires the use of specimen, treatment and time-point identifiers as well as other experimental attributes (data formats, alternative perspectives, transformations of the data such as changes in units, etc.) to be captured in such a way as to preserve the main experimental relationships. In that case, we can expand our set of organisational resources to include file formats in addition to the set of symbols (letters, numerals, punctuation, uppercase and lowercase) used to create the various identifiers. Ideally, we would like to keep repetition to a minimum so that the nature of the experiment can be readily discerned.

The way organisational resources are used affects the usability of the resulting organisation: using too few of them will obscure the meaning of the organisation while using too many will overwhelm potential users. For example, including redundant folders along any part of the hierarchy (folders that contain only a single folder which in turn contains the actual data) makes it tedious to navigate through a dataset. On the other hand, dumping all files into one folder will make it difficult for the end user to distinguish between groups of semantically related files, especially when thousands of files are present. Similarly, naming files and folders by referring to entities inaccessible to their intended users (e.g., local machine names or private accession codes that external users will not have access to or even fathom) consumes precious ‘name space’ without conveying any useful information. Organising data is thus an investment of time and effort with the aim of improving the usefulness of the data.

We can therefore formulate the organisation task as follows: *given a set of related data items associated with an experiment, how may they be organised to best convey their relationships using as few organisational resources as possible while maximising their usability?*

To achieve this, we define the term *facet* to refer to the various attributes germane to the experiment which may be included in the folder and file names. A non-exhaustive list of facets is: specimen names ( *organism, tissue, cell type/line*), experimental roles ( *treatments vs. controls*), time ( *developmental status, date, elapsed time*), processing status ( *raw data, by algorithm, procedure*), commonly available experimental equipment ( *microscopes, detectors, preparation equipment model names*), replicates, file types ( *3D volumes, particle stacks*), names of software used for processing.

This guide attempts to solve the organisation task by providing 10 recommendations that arise from our experience of handling hundreds of large image datasets in the public archives EMPIAR, BioImage Archive and BioStudies ( Sarkans *et al.*, 2018). Ideally, we would like to organise potentially numerous and voluminous data to maximise ease of use and hence facilitate the user’s ability to:

1. quickly identify the suitability of (subsets of) the data;
2. clearly distinguish between the various facets of the data;
3. quickly verify the usefulness of the data (e.g., thumbnails, previews, summaries, READMEs, LICENCE files);
4. retrieve only relevant subsets of the data.

This guide does not offer any recommendations for a detailed schema to describe experimental and analytical procedures; those may be captured in metadata for the various archives. Neither does it describe how to decide which experimental facets are appropriate (these are part of the experimental design), nor does it attempt to describe how to achieve organisation for automated analysis (we assume that the resulting organisation will be consumed by humans). It also ignores the universe of image formats in use and mainly includes examples from our experience archiving bioimaging data, but we anticipate it may be useful across other imaging disciplines. Good organisation improves data structure and format predictability and may facilitate automated processing. Therefore, our guide is intended to lead towards best practices rather than serve as a framework. Finally, this guide does not aim to achieve standardisation. We believe it is more practical to have a set of best practices and leave it up to the data authors to decide how best to apply them.

We believe that the recommendations outlined here may be of value to two principal groups of users: 1) data depositors, who need to design and prepare their data to improve its usability to the community, and 2) technologists (hardware, software and methods developers), who, by considering these recommendations in their designs, can greatly facilitate good data organisation at the source.

## Recommendations

We will motivate our guide by referring to a fictitious EMPIAR dataset. This dataset has a clear structure, but we propose that it can be further improved following the recommendations in the guide below.

**Figure 1.**
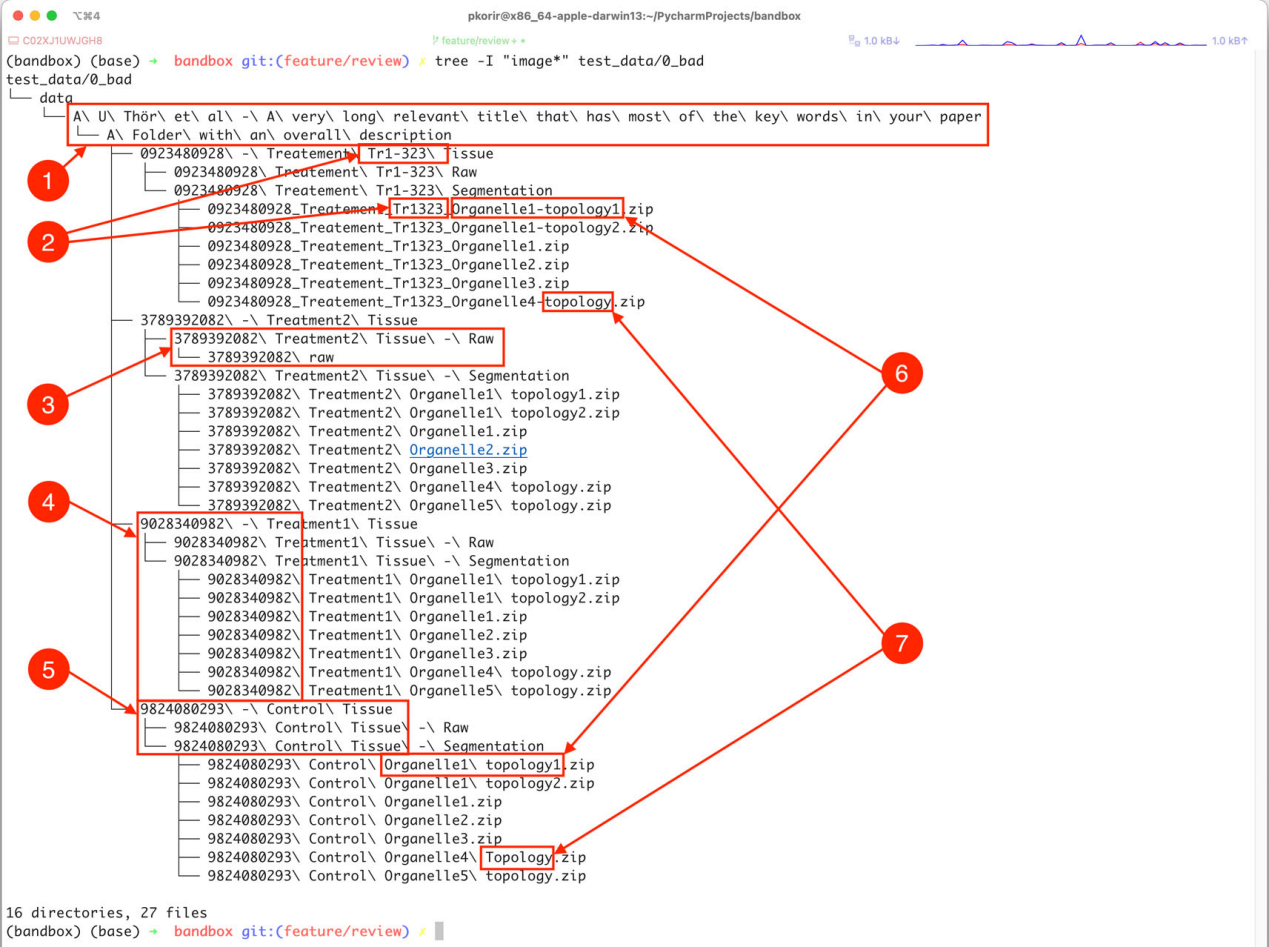
Illustration of some of the ways in which subtle aspects of data organisation impact its usability. These include: 1) long file/folder names with spaces, non-ASCII characters (ö) and redundant directories (ASCII – American Standard Code for Information Interchange); 2) obscure names or identifiers, possibly with inconsistent spelling, 3) inconsistency in folder hierarchy, 4) obscurity through meaningless names or identifiers, 5) verbosity in names, 6) subtle differences in spelling (in this case, a hyphen) and 7) inconsistency in typography due to character case and inclusion of different separator characters, e.g., spaces vs hyphens. See text for more details.

Our goal is to improve the file/folder structure shown in Figure 1 to better convey the relationships between the experimental facets while economising on the organisational resources available. For clarity, we have refrained from listing several thousand uncompressed TIFF files in the folders designated ‘Raw’ .

The example dataset illustrates several properties of its organisation that undermine the goal of being usable:

• **Verbosity/redundancy** typically manifested in repetition of references which may be resolved using the file hierarchy, such as:
  ○ Folders containing only a single folder which in turn contains the folder with the actual data. The child folder of ‘data’ only has the folder ‘A U Thör et al …’ in it that contains the folder ‘A folder with an overall description’ which has the actual data.
  ○ Very long names of files/folders. The full path of the file ‘data/A U Thör et al - A very long relevant title that has most of the keywords in your paper/A Folder with an overall description/0923480928 - Treatement Tr1-323 Tissue/0923480928_Treatement_Tr1323_Organelle1-topology1.zip’ is ‘0923480928 Treatement Tr1-323 Segmentation/0923480928_Treatement_Tr1323_Organelle1-topology1.zip’, which might be outside the limits of legacy software; e.g., IMOD ( Mastronarde, 2006) has a limit of 320 characters for input file names.
  ○ Repetition of identifiers along the path. In the previous example, half of the files repeat the identifier ‘0923480928’ that conveys no meaningful information and which, if required at all, should only appear in the appropriate parent folder name.
• **Ambiguity** occurs through incomplete identifiers either due to typos or non-standard characters.
  ○ Is ‘Tr1-323’ the same as ‘Tr1323’ ?
  ○ Use of spaces and non-ASCII characters can make processing the data complicated because of how software may handle path names with spaces. ASCII stands for the American Standard Code for Information Interchange and consists of plain characters used in many languages.
• **Inconsistency** is perhaps the most common issue and is usually the result of manually introduced errors such as changes in spelling, e.g., naming similar folders ‘tomo’ and ‘tomogram’ for related files. In the above example we have:
  ○
‘Topology’ and ‘topology’

  ○
‘Treatment’ vs ‘Treatement’

  ○
‘Tr1-323’ and ‘Tr1323’

  ○ Inconsistency may also be observed in folder structure. For example, only one of the treatment folders (the one with ‘3738932082’ in the name) has an extra child folder, breaking the pattern of the others.
• **Obscurity** tends to occur by identifiers with no obvious meaning, e.g., references to external resources such as figure numbers in a related paper, machine identifiers, script names, etc.
  ○ The numerical identifiers such as ‘0923480928’ have no obvious meaning in the context of the dataset.
  ○
‘Tr1-323’ may be an external reference but its meaning is unclear.
  Understandably, in certain cases such obscurity may be useful to keep identifiers which convey additional information. For example, in cryogenic-specimen electron microscopy (cryoEM) pipelines, the dataset may consist of multiple subsets obtained with different open-source software, e.g. particle picking by EMAN2 ( Tang *et al.*, 2007), beam-induced motion-correction by MotionCorr ( Li *et al.*, 2013), contrast-transfer function (CTF) correction by gCTF ( Zhang, 2016), classification by RELION ( Scheres, 2012), reconstruction by cryoSPARC ( Punjani *et al.*, 2017), etc.

The 10 recommendations we present below are divided into four groups: *planning* (recommendation 1), *structure* (recommendations 2-4), *naming* (recommendations 5-7) and *miscellaneous* (recommendations 8-10). We have provided further guidance within each group for related concepts.

### Planning

**(1) Design before data collection.** Plan beforehand, if possible, how the data will be structured.

a. If the experimental facets are known prior to data collection, the organisation suggestions that follow below will be easier to apply once and for all; it is harder to reorganise data after collection, especially voluminous data on multiple networked drives or in a cloud resource. At a minimum, consider organising the few top-level directories in terms of the experimental facets prior to archival.
b. Employ a naming convention within a research group or facility to ensure that data is consistent between data creators. This can even be specified in the microscope’s software to include imaging parameters in the file names automatically such as a base name, date and/or time, imaging parameters (e.g., resolution, section size) or even free text, among many others. We invite software vendors/creators that have not already done so to consider taking these recommendations into account.

### Structure

This section contains recommendations to address the hierarchical organisation of files and folders only.

**(2) Top-level folder.** Have one parent folder into which all sub-datasets are located. Such a top-level folder is also a good location to include auxiliary data that apply to the collection such as README or integrity verification files (see recommendation 10), which provide users with the context of the data organisation.

**(3) Filename length, path length and folder depth.**

a. Impose an upper limit on the length of file and folder names. We propose a working upper limit of 50 characters. Even though modern operating systems have no limitations on the lengths of names, end users will still struggle typing very long names which increases the likelihood of transcription errors. In some cases, older software that is still widely used by the bioimaging community imposes limits on the number of characters for file paths, e.g., IMOD ( Mastronarde, 2006) imposes a file-path length limit of 320 characters. It is useful to bear in mind that, increasingly, users interact with datasets via a web browser, which also has a practical limit (based on the device’s memory) on the number of files that can be selected in the browser's select dialog.
b. Limit the folder depth to a reasonable maximum. As a rule of thumb, three to four directory levels should be adequate for most applications but the fewer the better. This is in line with the ISA framework ( Sansone et al., 2008), which organises metadata in three levels (investigation, study, assay). In contrast, both shallow folder depth, with many and varied file types that are difficult to distinguish, and deep nesting of folders make navigation and selection a challenge.
c. Exclude intermediate levels of folders that do not convey any additional information. For example, consider a dataset having only TIFF files. Including an additional folder called tiff in the path <condition>/tiff/files*.tif is redundant. By contrast, if the file format is important then <condition>/<format1>/<files_of_format1> and <condition>/<format2>/<files_of_format2> is meaningful.
d. Impose an upper limit on the number of files in a folder and if necessary split large directories so they do not contain more than a certain maximum number of files (e.g., 10,000). If, for instance, a folder contains one million files then it could instead be organised as a folder ( parent_folder ) with 100 sub-folders ( child00 to child99 ), each containing 10,000 files. This is important because different file systems have different tolerances for handling large numbers of files. For example, the Second Extended Filesystem (ext2) imposes a ‘soft’ limits of 10,000 files per directory because of the extra overhead when processing such large folders ( *The Second Extended Filesystem — The Linux Kernel Documentation* ). While modern file systems are capable of handling larger numbers of files, the re-usability of the data will increase when taking into account systems with more modest resources, such as web browsers that may need to list or process all files in a directory.

**(4) Folder contents.**

a. Group related files unless it is instrumental to keep them separated. For example, group files by specimen, filetype, experimental purpose (treatment, control), etc. It may be crucial to separate different data types into different folders (e.g., one for micrographs and one for particle stacks). Further sub-folders may be necessary for single- and multi-frame micrographs, unaligned and aligned micrographs, etc.
b. Deposit data from different experimental techniques/modalities as separate archive entries (e.g., single-particle cryoEM data in one, tomography data in another). Some archives allow multiple related but separate entries to be linked or grouped.

### Naming

In this section, we provide some suggestions to improve the naming of files and folders.

**(5) Meaningful names.**

a. Name files and folders using meaningful identifiers without specifying external references. For instance, while the name ‘Figure 5’ probably refers to a figure in a paper describing (some of) the data, users will require access to the article, which may be behind a paywall or in a hard-to-find book. The names of files and folders should exclude any identifiers indicating a particular instrument or your organisation.
b. Avoid ambiguous attributes such as dates and times particularly in folder names. Mass renaming of files with dates and times can become non-trivial particularly if such attributes vary subtly (e.g., date, minute, seconds) from file to file.

**(6) Naming symbols**.

a. Restrict names to numerals and lowercase letters and replace all spaces with underscores or hyphens for meaningful word (group) boundaries, to make it substantially easier to work with the data. This facilitates easy transition between typing command line utilities or program names, which invariably work with lowercase (Windows PowerShell cmdlets are case insensitive even though they are documented in CamelCase e.g. Get-Command; similarly, macOS path names are case insensitive by default, though this depends on the chosen file-system formatting). Use underscores only for word boundaries and hyphens for keywords or other key attributes such as specimen names identifiable by the presence of a hyphen, e.g., covid-19 . Consistent use of case also improves readability ( Deissenboeck & Pizka, 2006).
b. Avoid certain characters which could lead to unintended consequences during processing such as ampersands (&), spaces, exclamation marks (!) and question marks (?). In general, stick to the portable character set defined by POSIX and avoid non-ASCII characters (e.g., ü, å or non-Roman scripts) to improve usability. Most keyboards can produce them, and most users will be familiar with them from everyday use. Also, some software will not work with input filenames featuring non-POSIX characters.
c. Avoid periods in names as this can lead to unpredictable behaviour for instance when attempting to determine formats. For example, while it is generally well known that the file file.tar.gz has two standard extensions, it may not be as widely known that file.ome.tiff, file.ome.tf2, file.ome.tf8 and file.ome.btf are all valid multi-extension bioimaging formats ( *OME-TIFF Specification — OME Data Model and File Formats 6.2.2 Documentation* ).

**(7) Identity**.

a. Ensure consistency when naming different files and folders related to one another. For example, in Figure 1, labels 6 and 7 show subtle changes in spelling or inclusion/exclusion of characters, which break the naming pattern.
b. Do not include personal identifiers (e.g., usernames, actual names, etc.) in folder or file names.
c. Some words to consider for exclusion in the names of files and folders: ‘files’ , ‘data’ , ‘images’ etc. as well as or other words that convey no additional meaningful information.
d. Think of folder names as applying to all the folders and files they contain as well: there should be no repetition in nested folder names, e.g., data/control.a/control.a.1/control.a.1.value/data/ ;
e. When providing 3D data as slices or sequentially ordering files, zero-pad the slice/file identifiers correctly (e.g. prefix-0099.tif not prefix-99.tif for thousands of slices), which guarantees that slices are correctly ordered lexicographically. Failing to do so could result in files being processed in the wrong order and e.g. lead to 3D stacks with misplaced slices, which will affect all analysis steps that follow. For example, consider a volume consisting of 1000 images, each of dimension M by N. Splitting this file should result in file names of the form file 0001.tif to file 1000.tif . Incorrect names can be fixed using the rename shell utility, e.g., rename file file 00 file??.tif will convert all files with 01 to 99 to have 0001 to 0099 . rename is available on most Linux distributions and may be installed on macOS using Homebrew or from the source code. On Windows systems the *Bulk Rename Utility* can be used.

### Miscellaneous

Finally, this section includes some tips on how to handle other aspects of organisation not covered in the previous sections.

**(8) File formats.**

a. Provide images in widely used file formats unless you are demonstrating a novel file format in which case it may be necessary to first get in touch with the archive to plan accordingly. Additional information may be requested to provide users with guidelines on how to use and visualise the new format files including any conversion tools that are available or providing the same data in a widely used file format as well.
b. Even for file types that are widely used, stick to open formats to ensure that users without access to proprietary software can access the data. Open formats promote the prevalence of tools (open source or proprietary) that can read and write data. We recommend the use of OME-NGFF ( Moore *et al.*, 2021) and OME-TIFF ( Linkert *et al.*, 2010) as open, widely supported imaging file formats.

**(9) Document your data.**

a. Include a README text file which provides an overview of how the data is organised. Depositors may use it to discover the main facets by which the data is organised, the structure of any ad hoc text files as well as the meaning of naming entities used in file/folder names.
b. Test the usability of your data by asking a colleague to peruse your data to assess whether the organisation is clear.

**(10) Integrity.** Include checksums, parity codes or hashes for each data file in a separate file, e.g., md5-sums.txt , imageset01.par2 or sha512-hashes.txt to facilitate content verification. These will allow users to verify that the data has not been corrupted during the deposition or download process. Each of these different ways to verify file integrity have corresponding tools available for all operating systems, but their operation is beyond the scope of this article ( Lianhua & Xingquan, 2017).

**Figure 2.**
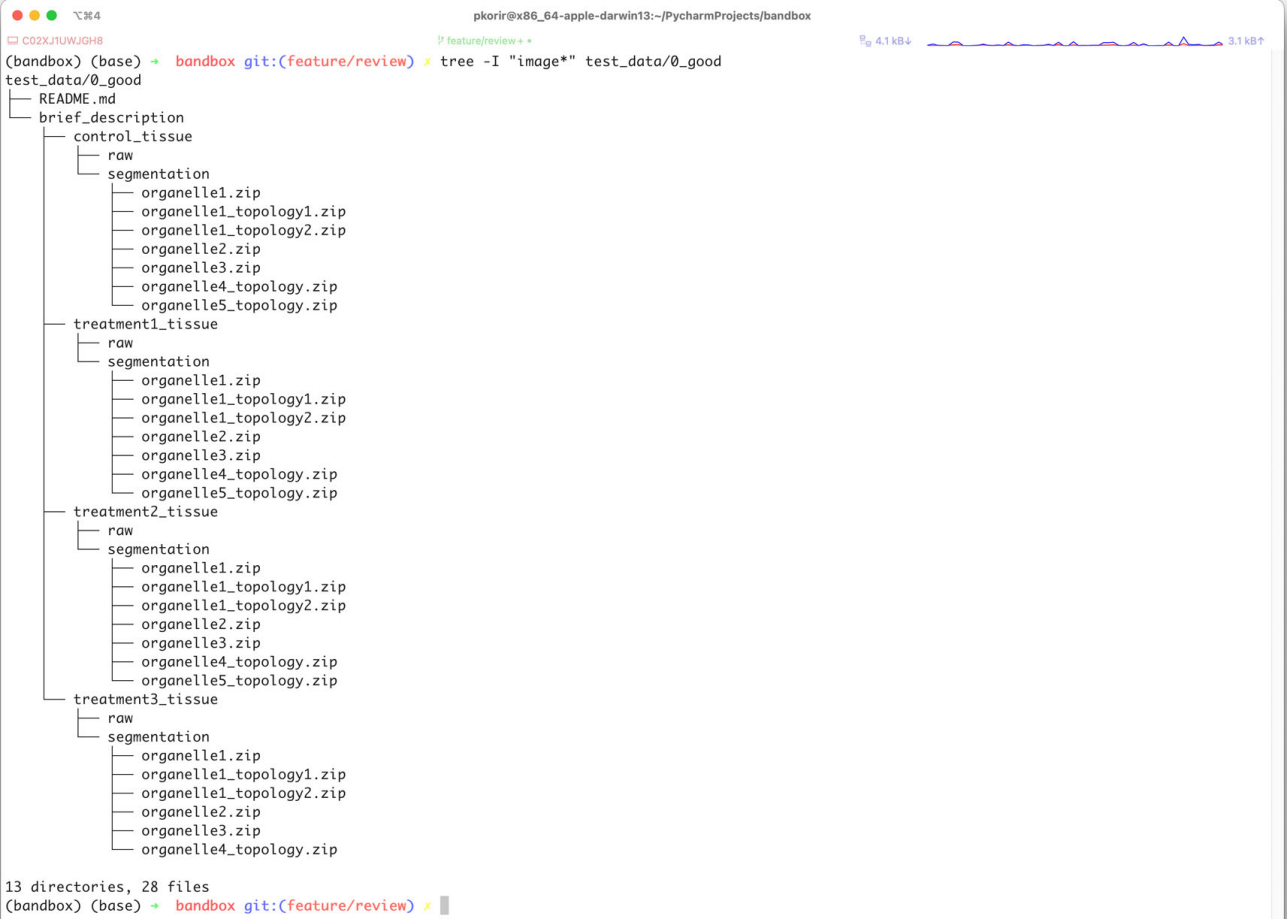
Tree representation of the data from Figure 1 reorganised by applying some of the 10 recommendations described in this paper.

Applying the recommendations above, we may revise the path:

data/A U Thör et al - A very long relevant title that has most of the keywords in your paper/A Folder with an overall description/0923480928 - Treatement Tr1-323 Tissue/0923480928_Treatement_Tr1323_Organelle1-topology1.zip’ is ‘0923480928 Treatement Tr1-323 Segmentation/0923480928_Treatement_Tr1323_Organelle1-topology1.zip

to:

data/brief_description/treatment3_tissue/segmentation/organelle1_topology1.zip

to achieve a reduction from 328 to 79 characters for the full path. The new organisation is presented in Figure 2.

## Conclusion

We hope that these 10 recommendations will only be the beginning of a broader discussion on how to organise bioimaging data in particular and experimental data in general for maximum usefulness, not just to the bioimaging community, but to the wider scientific community. Given the breadth of applications of bioimaging techniques, good organisation would go a long way to helping scientists from other disciplines to benefit from using bioimaging data. There is still considerable scope to develop better ways of not only organising data, but also representing it to enable automated data analysis.

## Figures and Tables

**Figure 3.**
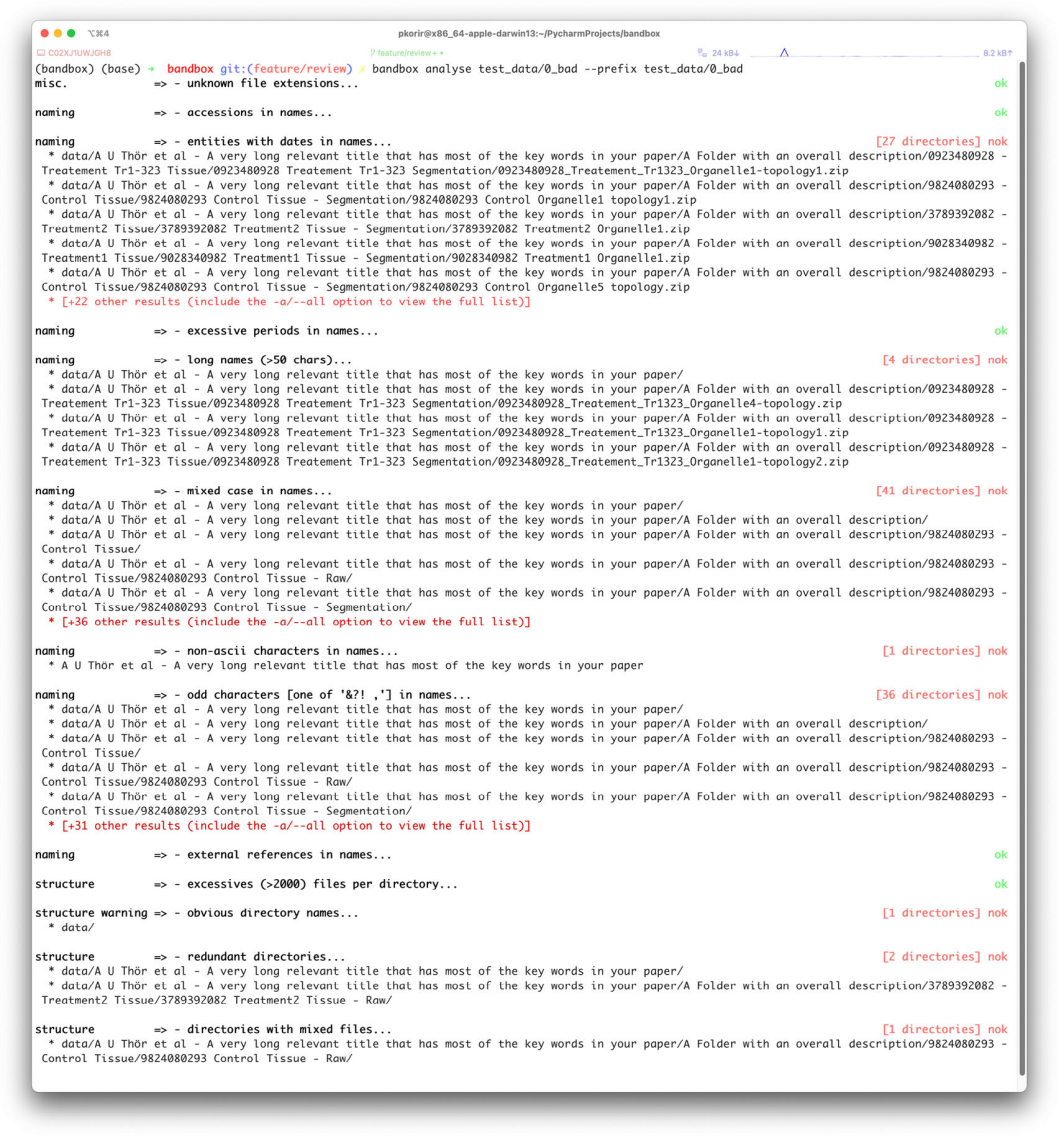
Example of a dataset with organisational issues identified by bandbox such as use of spaces or non-ASCII characters, redundant directories and other categories with an indication of the number of such entities found in each category. The example dataset is provided with the bandbox source code (ASCII – American Standard Code for Information Interchange).

**Figure 4.**
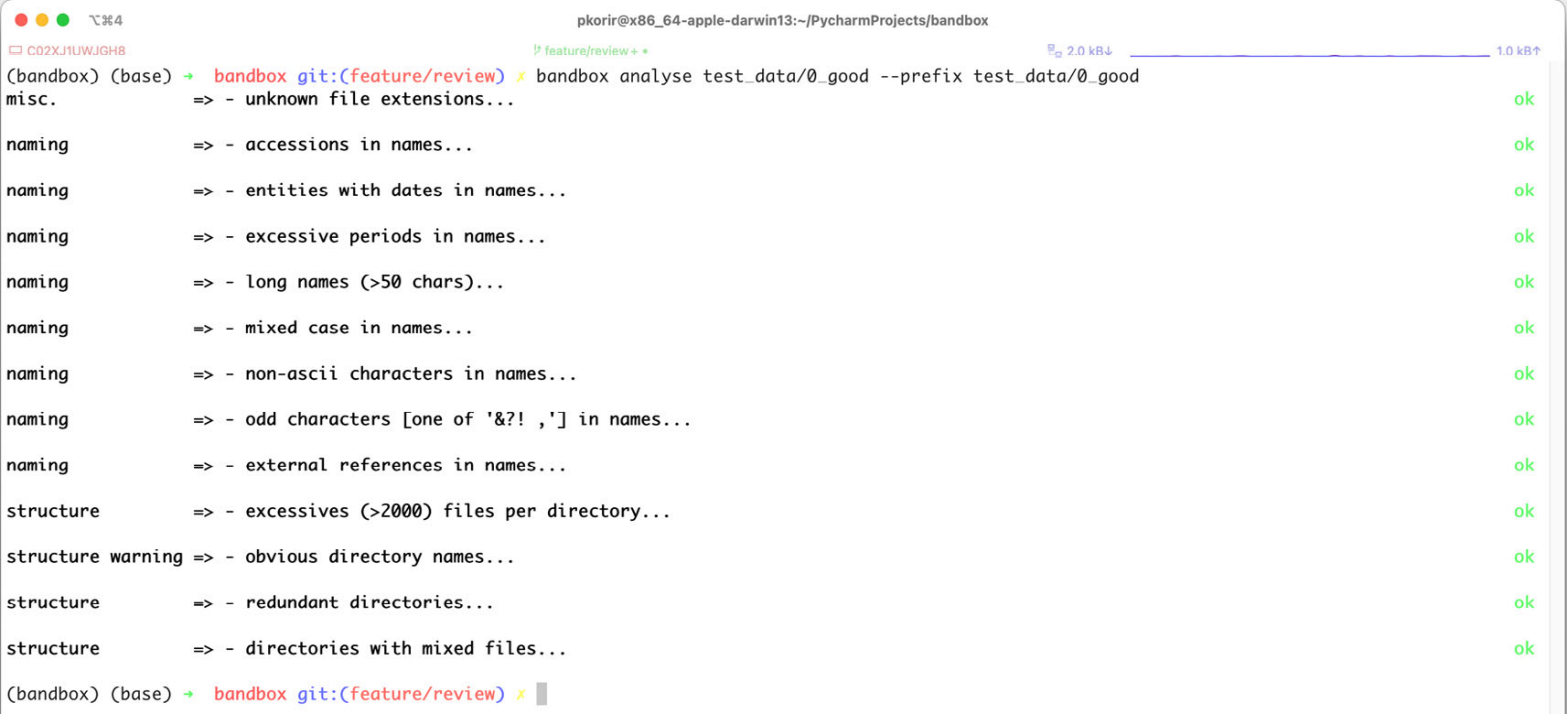
Example of a dataset with no issues identified by bandbox . The example dataset is provided with the bandbox source code.

## Data Availability

No data are associated with this article.

## Software availability

To make our recommendations practical, we have developed bandbox ( Korir *et al.*, 2022), an open-source command-line interface (CLI) tool to help users understand how they can improve the organisation of their data in preparation for archival. The program offers two CLI commands: view and analyse. Running bandbox view <dir> command displays a tree of a directory and all its contents; for every non-empty directory with files, bandbox provides a summary of the number of files in it, including a list of all the file formats encountered. Running bandbox analyse <dir> command provides a listing of possible issues grouped into categories in line with those specified in the Recommendations section. bandbox examines the tree associated with the nested hierarchy of files and folders in a dataset and then concurrently runs various heuristics on the tree which are controlled by configurations that the user may modify. The results produced by the analyse command are only suggestions for improvement; we understand that there may be practical limitations to implementing some of the suggested improvements as well as good reasons for keeping the data as is. We have designed bandbox to be configurable and extensible allowing users to customise analysis parameters (file/folder name length, recognised file formats, accession names, regexes) as well as add new heuristics. An example configuration file is provided in the Github repository. **Figures 3** **and** **4** show screenshots of the results of running bandbox on two different datasets.

Software available from: https://pypi.org/project/bandbox

Source code available from: https://github.com/emdb-empiar/bandbox

Archived source code at time of publication: https://doi.org/10.5281/zenodo.7807541 ( Korir *et al.*, 2022).

License: Apache License 2.0
