# Supplementary material for: Ten recommendations for organising bioimaging data for archival
Source: F1000Res. 2024 Feb 27;12:ELIXIR-1391. Originally published 2023 Oct 23. [Version 2] doi: 10.12688/f1000research.129720.2 (PMC10938051; doi:10.12688/f1000research.129720.2)
Supplement: Peer review history

# Reviewer report: Reviewer response for version 2

**DOI:** 10.5256/f1000research.162090.r250664

**Published:** 2024-05-09

**Authors:** William T. Katz, Virginia Scarlett

**Affiliations:** 1 Howard Hughes Medical Institute’s Janelia Research Campus, Ashburn, USA

We feel that the article remains at the status of 'Approved with Reservations'. We are grateful to the authors for the revisions that have been implemented, including a clearer explanation of 'organisational resources', improved readability of the figures, and a limit on the output of the bandbox program. However, the article references REMBI but not QUAREP-LiMi, and while it cites OME projects (OME-NGFF and OME-TIFF), it does not mention the impacts or limitations of those important efforts. Also, the article remains vague with respect to its audience. The authors should clarify to which biomedical imaging storage approaches (e.g., flat files, chunked formats, cloud-based APIs, etc.) their recommendations and tooling apply. If the article is intended for users of flat formats (such as TIFF) working on file systems, then the authors should clarify this because the title suggests a very broad applicability to archiving bioimaging data.

Is the topic of the opinion article discussed accurately in the context of the current literature?

Yes

Are arguments sufficiently supported by evidence from the published literature?

Partly

Are all factual statements correct and adequately supported by citations?

Partly

Are the conclusions drawn balanced and justified on the basis of the presented arguments?

Yes

Reviewer Expertise:

Data engineering; biomedical image processing and analysis.

We confirm that we have read this submission and believe that we have an appropriate level of expertise to confirm that it is of an acceptable scientific standard, however we have significant reservations, as outlined above.

# Reviewer report: Reviewer response for version 2

**DOI:** 10.5256/f1000research.162090.r250665

**Published:** 2024-04-04

**Authors:** Kenneth H. L. Ho

**Affiliations:** 1 Advanced Light Microscopy, The Francis Crick Institute, London, England, UK

The authors have addressed all my previous comments, and I am satisfied with their revision. I have no further comments to make..

Is the topic of the opinion article discussed accurately in the context of the current literature?

Yes

Are arguments sufficiently supported by evidence from the published literature?

Partly

Are all factual statements correct and adequately supported by citations?

Yes

Are the conclusions drawn balanced and justified on the basis of the presented arguments?

Yes

Reviewer Expertise:

Bioimage informatics

I confirm that I have read this submission and believe that I have an appropriate level of expertise to confirm that it is of an acceptable scientific standard.

# Reviewer report: Reviewer response for version 2

**DOI:** 10.5256/f1000research.162090.r250663

**Published:** 2024-03-18

**Authors:** Sylvia Emmanuelle Le Dévédec

**Affiliations:** 1 Division of Drug Discovery and Safety, Leiden Academic Centre of Drug Research, Universiteit Leiden, Leiden, South Holland, The Netherlands

The revised version has effectively addressed the majority of my comments. Clarity regarding the intended audience and the importance of data archiving has been improved, and ambiguities have been identified and addressed.

Is the topic of the opinion article discussed accurately in the context of the current literature?

Partly

Are arguments sufficiently supported by evidence from the published literature?

Partly

Are all factual statements correct and adequately supported by citations?

Yes

Are the conclusions drawn balanced and justified on the basis of the presented arguments?

Yes

Reviewer Expertise:

Biology; image-based phenotypic profiling; microscopist; data generator;  core facility management; FAIR metadata

I confirm that I have read this submission and believe that I have an appropriate level of expertise to confirm that it is of an acceptable scientific standard.

# Reviewer report: Reviewer response for version 2

**DOI:** 10.5256/f1000research.162090.r250662

**Published:** 2024-03-13

**Authors:** Sjors Scheres

**Affiliations:** 1 Medical Research Council Laboratory of Molecular Biology, Cambridge, England, UK

Most of my comments have been addressed in the revised version.

Is the topic of the opinion article discussed accurately in the context of the current literature?

Yes

Are arguments sufficiently supported by evidence from the published literature?

Yes

Are all factual statements correct and adequately supported by citations?

Partly

Are the conclusions drawn balanced and justified on the basis of the presented arguments?

Partly

Reviewer Expertise:

Structural biologist; software developer

I confirm that I have read this submission and believe that I have an appropriate level of expertise to confirm that it is of an acceptable scientific standard.

# Reviewer report: Reviewer response for version 1

**DOI:** 10.5256/f1000research.142422.r217552

**Published:** 2023-11-21

**Authors:** William T. Katz, Virginia Scarlett

**Affiliations:** 1 Howard Hughes Medical Institute’s Janelia Research Campus, Ashburn, USA

In this opinion article, the authors tackle an important but often-overlooked aspect of biomedical data archives: how best to organize data folders and files to maximize ease of use. Ten recommendations are provided as well as a lightweight command-line tool for inspecting datasets. Since there continues to be an acceleration in both the number and size of these datasets accessible through various repositories, both the recommendations and tool from experienced archivists are useful and should be published, though we feel some revision of the document is warranted.

 The introduction describes the broader context of bioimaging data management before focusing on the contributions of the article.  There could be clearer differentiation of efforts to standardize bioimaging metadata (REMBI, QUAREP-LiMi), file formats and associated libraries (OME-TIFF, OME-NGFF, Zarr, n5), and local or cloud-based services that provide Data APIs (DVID, BossDB) with some level of abstraction in how data is actually stored. The recommendations and tool mainly apply to file-based solutions though some of the recommendations, such as naming, would be applicable to other forms of big data repositories. We suggest that the authors clarify the scope of their contributions.

 It should be noted that some of the efforts to standardize data and its distribution also have recommendations for organization of data. For example, OME-NGFF requires segmentation to be in a directory called “labels/”.

 In the third paragraph of Motivation, the terms “ways and means” and “organisational resources” are unclear though some of your examples (folder hierarchy, file formats, identifiers) show how data can be organized. We suggest you start with some examples and then introduce “organizational resources” as a term.

 If standardization is not an aim, can bandbox be configured to remove warnings not agreed upon by a user?  In Figure 2, the printing of the word “warning” for datasets with no red flags seems odd.  We would suggest using “check” as in “name check” or “structure check” if no warnings exist.

 Given recommendation (8)b and the article’s bioimaging focus, the bandbox tool should work by default with well-known, large-scale formats like Zarr and N5.  In testing, it appears that bandbox doesn’t recognized file extensions used by such formats like .json and .zarr. The configurability of bandbox is a nice feature and should be mentioned in the article. This would allow other tool builders to contribute configurations for validating common formats and it seems like the regex capability could allow folder hierarchy requirements.

 The command-line bandbox tool should limit warning output to some maximum number of lines by default. This is particularly true for massive, chunked datasets consisting of many files and folders. We would suggest adding a “verbose” flag to allow full results to be output perhaps to a file.

 Some minor points:

 The description of the bandbox tool could be moved out of the Motivation section and after listing the recommendations.

 Figure 1 has too small font sizes and would not be readable for printed copies as well as expending quite a bit of black ink.

 The phenomenon described in the first sub-bullet under 'Verbosity' is an interesting point that seems to deserve its own name. Maybe something like, 'redundant nesting' or 'over-nesting'. A name would also make it easier to connect to solution 3A, which is conceptually related. Also, the second half of this bullet point is in monospace font, but it should be Times New Roman or whatever.

 Some recommendations are more universally advisable than others. For those points, we’d recommend dropping “Consider” for stronger language.

 In (4)b, “Most archives allow multiple separate entries to be linked or grouped,” it’s not clear what qualifies as an archive since data could be made available through cloud providers’ object stores and other facilities.

 In (5)b, could you clarify in what ways dates and times are “ambiguous attributes”?

 In the sentence, “bandbox examines the tree associate with the nested hierarchy…” the word bandbox should be in monospace font.

 What is the rationale for limiting folder depth to 3 or 4 levels?

 In (7)b, “Do not include personal identifiers in folder names.” Personal identifiers should be clarified.

 For (7)e, zero-padding should be considered for any sequentially ordered set of files. A good case is 2D slices of a 3D volume as described.

 For (8)b, consider citing OME-NGFF and OME-TIFF as recommended community formats.

 For (9)a, the recommendation for an overview could explicitly suggest listing the facets used to organize the data.

 In Figure 4, is the single “brief_description” folder at that level recommended instead of adding the descriptive information to a README file?  Perhaps a real description should be used in the example to make it clear why recommendation (3)b doesn’t apply.

Is the topic of the opinion article discussed accurately in the context of the current literature?

Yes

Are arguments sufficiently supported by evidence from the published literature?

Partly

Are all factual statements correct and adequately supported by citations?

Partly

Are the conclusions drawn balanced and justified on the basis of the presented arguments?

Yes

Reviewer Expertise:

Data engineering; biomedical image processing and analysis.

We confirm that we have read this submission and believe that we have an appropriate level of expertise to confirm that it is of an acceptable scientific standard, however we have significant reservations, as outlined above.

# Response

**Published:** 2024-01-30

**Authors:** Gerard J Kleywegt

**Affiliations:** EMBL European Bioinformatics Institute, UK

**Response to Reviewer #4** **(in italics)**

 Specific comments:

 In this opinion article, the authors tackle an important but often-overlooked aspect of biomedical data archives: how best to organize data folders and files to maximize ease of use. Ten recommendations are provided as well as a lightweight command-line tool for inspecting datasets. Since there continues to be an acceleration in both the number and size of these datasets accessible through various repositories, both the recommendations and tool from experienced archivists are useful and should be published, though we feel some revision of the document is warranted.

 The introduction describes the broader context of bioimaging data management before focusing on the contributions of the article.  There could be clearer differentiation of efforts to standardize bioimaging metadata (REMBI, QUAREP-LiMi), file formats and associated libraries (OME-TIFF, OME-NGFF, Zarr, n5), and local or cloud-based services that provide Data APIs (DVID, BossDB) with some level of abstraction in how data is actually stored. The recommendations and tool mainly apply to file-based solutions though some of the recommendations, such as naming, would be applicable to other forms of big data repositories. We suggest that the authors clarify the scope of their contributions.

 It should be noted that some of the efforts to standardize data and its distribution also have recommendations for organization of data. For example, OME-NGFF requires segmentation to be in a directory called “labels/”.

 In the third paragraph of Motivation, the terms “ways and means” and “organisational resources” are unclear though some of your examples (folder hierarchy, file formats, identifiers) show how data can be organized. We suggest you start with some examples and then introduce “organizational resources” as a term.

*We accept this correction and have updated the text to better reflect this point.*

 If standardization is not an aim, can bandbox be configured to remove warnings not agreed upon by a user?  In Figure 2, the printing of the word “warning” for datasets with no red flags seems odd.  We would suggest using “check” as in “name check” or “structure check” if no warnings exist.

*We have released an updated version (bandbox v0.2.2) where these have been amended.*

 Given recommendation (8)b and the article’s bioimaging focus, the bandbox tool should work by default with well-known, large-scale formats like Zarr and N5.  In testing, it appears that bandbox doesn’t recognized file extensions used by such formats like .json and .zarr. The configurability of bandbox is a nice feature and should be mentioned in the article. This would allow other tool builders to contribute configurations for validating common formats and it seems like the regex capability could allow folder hierarchy requirements.

*We have clarified in the text that bandbox is configurable.*

 The command-line bandbox tool should limit warning output to some maximum number of lines by default. This is particularly true for massive, chunked datasets consisting of many files and folders. We would suggest adding a “verbose” flag to allow full results to be output perhaps to a file.

*This has been updated in bandbox v0.2.2. Instead of printing all results by default, we have substituted the* *-S/--summarise flag with a* *-a/--all flag so that by default users don’t get overwhelmed. The instruction to use the new flag is now highlighted in yellow text beneath each section with more than a certain number of results.*

 Some minor points:

 The description of the bandbox tool could be moved out of the Motivation section and after listing the recommendations.

*We have now included a detailed description of bandbox in the Software Availability section.*

 Figure 1 has too small font sizes and would not be readable for printed copies as well as expending quite a bit of black ink.

*We accept the suggestion and have changed all images to have a light background.*

 The phenomenon described in the first sub-bullet under 'Verbosity' is an interesting point that seems to deserve its own name. Maybe something like, 'redundant nesting' or 'over-nesting'. A name would also make it easier to connect to solution 3A, which is conceptually related. Also, the second half of this bullet point is in monospace font, but it should be Times New Roman or whatever.

*We have given the section the name ‘Verbosity/Redundancy’.*

*The use of monospace font here is intentional to distinguish between literal text and computer text (file/folder names, commands, tools).*

 Some recommendations are more universally advisable than others. For those points, we’d recommend dropping “Consider” for stronger language.

 In (4)b, “Most archives allow multiple separate entries to be linked or grouped,” it’s not clear what qualifies as an archive since data could be made available through cloud providers’ object stores and other facilities.

*We have provided a definition of ‘archive’ in the opening paragraph of the article.*

 In (5)b, could you clarify in what ways dates and times are “ambiguous attributes”?

*We have provided an explanation on this in the text.*

*Dates and times are ambiguous to the extent that they do not provide meaningful attributes associated with the experiment. While it can be assumed that dates on file names refer to the date of collection, this is not instrumental to the actual data i.e. knowing the date of collection adds no scientific value. Furthermore, having every single image file with the same date consumes precious ‘naming space’ of files, which can either be provided once in the name of the parent folder or as part of the metadata, where it would be expected to convey useful information to users.*

 In the sentence, “bandbox examines the tree associate with the nested hierarchy…” the word bandbox should be in monospace font.

*This has been corrected in the text.*

 What is the rationale for limiting folder depth to 3 or 4 levels?

*We have argued this point based on the ISA framework.*

 In (7)b, “Do not include personal identifiers in folder names.” Personal identifiers should be clarified.

*We accept this point and have provided some examples of what is meant by ‘personal identifiers’.*

 For (7)e, zero-padding should be considered for any sequentially ordered set of files. A good case is 2D slices of a 3D volume as described.

*We have included ‘sequential ordering’ as another example of this phenomenon.*

 For (8)b, consider citing OME-NGFF and OME-TIFF as recommended community formats.

*We accept the suggestion and have amended the text as requested.*

 For (9)a, the recommendation for an overview could explicitly suggest listing the facets used to organize the data.

*We have included a sentence outlining what may be included in the README file.*

 In Figure 4, is the single “brief_description” folder at that level recommended instead of adding the descriptive information to a README file?  Perhaps a real description should be used in the example to make it clear why recommendation (3)b doesn’t apply.

*This term is purely illustrative as are the names of the files and folders.*

# Reviewer report: Reviewer response for version 1

**DOI:** 10.5256/f1000research.142422.r217556

**Published:** 2023-11-21

**Authors:** Sylvia Emmanuelle Le Dévédec

**Affiliations:** 1 Division of Drug Discovery and Safety, Leiden Academic Centre of Drug Research, Universiteit Leiden, Leiden, South Holland, The Netherlands

The guideline presented by Korir and colleagues, who are recognized experts in data structure, data organization, and FAIRification, marks an important step toward fostering a comprehensive discussion on the management of bioimaging data. The authors, primarily developers and bioinformaticians dealing with intricate datasets, have assembled a set of recommendations that, while valuable, may be perceived as overly abstract, potentially posing challenges for experimentalists who serve as the primary data producers.

 One critical aspect that emerges is the need for greater clarity regarding the intended audience for this guideline. Currently, it appears somewhat ambiguous, leading to potential misalignment with the individuals it should be primarily targeting. It is recommended that the authors explicitly define the community they aim to address at the outset of the manuscript. If, indeed, the target audience is the data producers, particularly experimentalists, then a comprehensive revision of the recommendations may be necessary. Consideration should be given to conveying the guidelines in a more accessible language, ensuring that the practical implications for experimentalists are clearly delineated. Additionally, the authors might explore the possibility of tailoring specific sets of guidelines for distinct roles, such as data managers and data producers, to enhance relevance and utility.

 Below are listed some specific points of attentions:

Abstract:

- The abstract lacks clarity on the intended audience of these recommendations. It is essential to specify whether the guidelines primarily target core facility managers, data managers/stewards, bioinformaticians, or experimentalists.
- If the guidelines are intended for experimentalists, the current manuscript may not align with the needs of this non-expert audience. The language and content may need to be adapted to cater to individuals with limited knowledge in data management.
- The phrase "make future data depositions more useful" needs clarification. Who benefits from this increased usefulness, and in what way? Is the goal to enhance practicality, efficiency, or accessibility? A more specific explanation would enhance the abstract's clarity.
- The term "bioimaging community" is used in the abstract, but its specific meaning in the context of this manuscript is unclear. Defining this community will provide readers with a better understanding of the scope and relevance of the guidelines.
- The abstract mentions that Bandbox is designed "to facilitate the process of analyzing data organization." It would be beneficial to elaborate on how the analyzing functionality of Bandbox directly benefits the bioimaging community. Specific examples or scenarios demonstrating its advantages would enhance the abstract's informativeness.

 
Introduction  

- What does data ‘archiving’ means exactly in this specific manuscript?
- Objective of the guideline: Harmonising how to organise datasets for maximum usefulness with archival in mind?
- Where this organization should occur in the data life cycle: before/during or after generation? Where this organisation should occur? In which physical storage space?
- Organisation = order of the data. Organisation or order of the data should be implicitly connected to the related metadata and even contained somewhere in the metadata.
- ‘Good organisation (order) of data improves its usefulness and is the responsibility of the data depositors.’ Do you mean here the data generator or specifically the data depositor? Based on the description it seems like the data depositor is implicitly the data generator.
- ‘Users can immediately distinguish the various experimental categories’: should you not refer to (p)ISA to clarify what is meant by ‘experimental categories’ (https://doi.org/10.1038/s41597-022-01805-5)?
- ‘Facet refers to the various attributes germane to the experiment which may be included in the folder and file names’. Should ‘facet’ not be called ‘key’? If not then explain the differences between both terms.  

Recommendations:

  

- The potential users for these recommendations lack clear definition, and depending on the proposed users, the guide should be tailored for optimal understanding. Data depositors and generators often have different levels of familiarity compared to program developers or data stewards, employing distinct languages. Addressing these differences is crucial for ensuring accessibility and effectiveness.
- Open-source command-line interfaces can be intimidating, particularly for experimentalists who serve as the primary data generators and often act as data depositors. As a cell biologist and experimentalist, I find the proposed CLI tool, while impressive and useful, potentially challenging to navigate comfortably. Enhancements in user-friendliness or alternative interfaces might significantly benefit experimentalists who are integral to both data generation and deposition.
- Given the recommendation for data producers to pre-define structures before data collection, it becomes apparent that the target audience of this guide is experimentalists with limited knowledge of data management and programming. Including guidelines or tips on naming conventions would be particularly valuable for such users, enhancing the practicality and applicability of the recommendations.
- The suggestion regarding folder contents description appears somewhat vague and may not be universally suitable for various experiment types. A more nuanced approach that considers the diversity of experiments would enhance the guide's usability.
- The concept of "meaningful names" for folders raises questions about subjectivity and human sensitivity, which may not align with the precision required for effective data management structures. Establishing a clear naming convention, is objectively applicable across various contexts, would contribute to the robustness and reliability of the guide.

Is the topic of the opinion article discussed accurately in the context of the current literature?

Partly

Are arguments sufficiently supported by evidence from the published literature?

Partly

Are all factual statements correct and adequately supported by citations?

Yes

Are the conclusions drawn balanced and justified on the basis of the presented arguments?

Yes

Reviewer Expertise:

Biology; image-based phenotypic profiling; microscopist; data generator;  core facility management; FAIR metadata

I confirm that I have read this submission and believe that I have an appropriate level of expertise to confirm that it is of an acceptable scientific standard, however I have significant reservations, as outlined above.

## References

[1] : pISA-tree - a data management framework for life science research projects using a standardised directory tree. *Scientific Data* .2022;9(1) : 10.1038/s41597-022-01805-5 10.1038/s41597-022-01805-5 36357404 PMC9649664

# Response

**Published:** 2024-01-30

**Authors:** Gerard J Kleywegt

**Affiliations:** EMBL European Bioinformatics Institute, UK

**Response to Reviewer #3** **(in italics)**

 Specific comments:

 The guideline presented by Korir and colleagues, who are recognized experts in data structure, data organization, and FAIRification, marks an important step toward fostering a comprehensive discussion on the management of bioimaging data. The authors, primarily developers and bioinformaticians dealing with intricate datasets, have assembled a set of recommendations that, while valuable, may be perceived as overly abstract, potentially posing challenges for experimentalists who serve as the primary data producers.

 One critical aspect that emerges is the need for greater clarity regarding the intended audience for this guideline. Currently, it appears somewhat ambiguous, leading to potential misalignment with the individuals it should be primarily targeting. It is recommended that the authors explicitly define the community they aim to address at the outset of the manuscript. If, indeed, the target audience is the data producers, particularly experimentalists, then a comprehensive revision of the recommendations may be necessary. Consideration should be given to conveying the guidelines in a more accessible language, ensuring that the practical implications for experimentalists are clearly delineated. Additionally, the authors might explore the possibility of tailoring specific sets of guidelines for distinct roles, such as data managers and data producers, to enhance relevance and utility.

 Below are listed some specific points of attentions:

Abstract:

- The abstract lacks clarity on the intended audience of these recommendations. It is essential to specify whether the guidelines primarily target core facility managers, data managers/stewards, bioinformaticians, or experimentalists.
*We welcome the suggestion to clarify the intended audience and have updated the abstract to clarify this.*
- If the guidelines are intended for experimentalists, the current manuscript may not align with the needs of this non-expert audience. The language and content may need to be adapted to cater to individuals with limited knowledge in data management.
*We aim to address a wide and varied audience, so the language and terminology needs to strike a balance for the content to be accessible and digestible by different groups. We hope we have managed a reasonable balance, especially following the many constructive suggestions of all the reviewers. If this reviewer has specific comments on sections in the revised manuscript that could be improved further in this respect we would be happy to attempt to do so.*
- The phrase "make future data depositions more useful" needs clarification. Who benefits from this increased usefulness, and in what way? Is the goal to enhance practicality, efficiency, or accessibility? A more specific explanation would enhance the abstract's clarity.
*We accept the correction and have spelled out in more precise terms what ‘more useful’ means and to whom this applies.*
- The term "bioimaging community" is used in the abstract, but its specific meaning in the context of this manuscript is unclear. Defining this community will provide readers with a better understanding of the scope and relevance of the guidelines.
*We accept the correction and have amended the text to reflect this.*
- The abstract mentions that Bandbox is designed "to facilitate the process of analyzing data organization." It would be beneficial to elaborate on how the analyzing functionality of Bandbox directly benefits the bioimaging community. Specific examples or scenarios demonstrating its advantages would enhance the abstract's informativeness.
*We accept the correction and have included, in the text, some examples of what bandbox is capable of doing.*

 
Introduction 

- What does data ‘archiving’ means exactly in this specific manuscript?
*We have included a definition of ‘archiving’ in the opening paragraph of the article.*
- Objective of the guideline: Harmonising how to organise datasets for maximum usefulness with archival in mind?
*Yes.*
- Where this organization should occur in the data life cycle: before/during or after generation? Where this organisation should occur? In which physical storage space?
*The earlier the better. Recommendation #1 (Design before data collection) highlights the impact of data planning before collection commences. The remaining recommendations outline various suggestions on how to improve the usability of the data. The organisation typically would happen on the storage device but can be done either through consoles or the appropriate graphical user interfaces.*
- Organisation = order of the data. Organisation or order of the data should be implicitly connected to the related metadata and even contained somewhere in the metadata.
*We have edited the text for clarity.*
- ‘Good organisation (order) of data improves its usefulness and is the responsibility of the data depositors.’ Do you mean here the data generator or specifically the data depositor? Based on the description it seems like the data depositor is implicitly the data generator.
*Data depositors’ here refers to the individual(s) responsible for making the submission to the archive (previously defined) and this may or may not be the generator of the data. In many cases, the depositor is familiar with the data because they performed the analyses implying familiarity with handling the data.*
- ‘Users can immediately distinguish the various experimental categories’: should you not refer to (p)ISA to clarify what is meant by ‘experimental categories’ ( https://doi.org/10.1038/s41597-022-01805-5)?
*We are grateful to the reviewer for pointing out this reference which is now referred to in the text.*
- ‘Facet refers to the various attributes germane to the experiment which may be included in the folder and file names’. Should ‘facet’ not be called ‘key’? If not then explain the differences between both terms. 
*We used the term ‘facet’ in the same sense as in multifaceted, implying that a dataset may be viewed from various perspectives to discern distinct properties much in the same way as a gem. The reviewer’s proposal of ‘key’ does not fit this sense.*

Recommendations:

  

- The potential users for these recommendations lack clear definition, and depending on the proposed users, the guide should be tailored for optimal understanding. Data depositors and generators often have different levels of familiarity compared to program developers or data stewards, employing distinct languages. Addressing these differences is crucial for ensuring accessibility and effectiveness.
*We have addressed the specificity of the audience in the amendments to the abstract (above).*
- Open-source command-line interfaces can be intimidating, particularly for experimentalists who serve as the primary data generators and often act as data depositors. As a cell biologist and experimentalist, I find the proposed CLI tool, while impressive and useful, potentially challenging to navigate comfortably. Enhancements in user-friendliness or alternative interfaces might significantly benefit experimentalists who are integral to both data generation and deposition.
*We accept this comment and are only constrained by our capacity to extend the CLI tool to achieve the desired usability.*
- Given the recommendation for data producers to pre-define structures before data collection, it becomes apparent that the target audience of this guide is experimentalists with limited knowledge of data management and programming. Including guidelines or tips on naming conventions would be particularly valuable for such users, enhancing the practicality and applicability of the recommendations.
*Recommendations 5, 6 and 7 go into considerable detail about what names to choose, which symbols to use in names and matters relating to identity. We are willing to revise any of the provided recommendations which remain unclear.*
- The suggestion regarding folder contents description appears somewhat vague and may not be universally suitable for various experiment types. A more nuanced approach that considers the diversity of experiments would enhance the guide's usability.
*We appreciate that the authorship of this article does not represent the universe of experimental methods in imaging. We do point out various facets that may be relevant but leave it up to depositors (generators) who are in the best position to judge which to use when structuring/naming folders. We also point out in the abstract that we offer these recommendations to start discussions in various data-rich communities.*
* *
- The concept of "meaningful names" for folders raises questions about subjectivity and human sensitivity, which may not align with the precision required for effective data management structures. Establishing a clear naming convention, is objectively applicable across various contexts, would contribute to the robustness and reliability of the guide.
*As stated above, we do not think it necessary to specify exactly how data should be organised given the vast variety of experiments that can be carried out. We do state in the article (Motivation, paragraph 8) that “...our guide is intended to lead towards best practices rather than serve as a framework. …this guide does not aim to achieve standardisation. We believe it is more practical to have a set of best practices and leave it up to the data authors to decide how best to apply them.”*

# Reviewer report: Reviewer response for version 1

**DOI:** 10.5256/f1000research.142422.r217555

**Published:** 2023-11-08

**Authors:** Kenneth H. L. Ho

**Affiliations:** 1 Advanced Light Microscopy, The Francis Crick Institute, London, England, UK

The article is timely as we are facing a deluge of bioimaging data with higher resolutions and automation. It is therefore an area that needs more discussions, sharing of good practices.

 I mostly agree with all the recommendations given, although I feel that the authors may need to make a good argument for some recommendations. Some choices seem arbitrary and I would like to see the rationale  behind them.

 After reading the paper, I am a bit confused by the article’s intended target audience.

 Is the article recommendation aimed at most of the biologists who archive their bioimaging data mainly for the purpose of peer review and references? Or is the article and recommendation aiming for those database curators and producers of bioimaging databases, e.g. IDR ( https://idr.openmicroscopy.org/) , SSBD ( https://ssbd.riken.jp/database/), GDC ( https://portal.gdc.cancer.gov/) , etc?

 On page 4 under the heading ‘Motivation’, “We believe that recommendations outlined here maybe of value to two principal groups of users: 1) data depositors, who need to design and prepare their data to improve its usability to the community”.

 Does it include most biologists? I believe that most biologists archive their data to provide a record of their studies. Are the data depositors in the article and its intended audience refer to bioimaging database curators/producers instead of bench biologists?

 I believe that the ten recommendations would be equally applied to most biologists even though their aim is to provide a record of their studies, the recommendations would help those database curators to organise their bioimage data in more meaningful ways.****

 I would like to see that part to be make clearer of its intended audience.

 With regards to the recommendations, on page 8, ‘Naming’ (5) Meaningful names (b) “Consider avoiding ambiguous attributes such as dates and times.

 The argument that they have “subtle variations” is not obvious to me.  Is it because of variations of date formats used in different countries? Would it be solved if ISO 8601 ( https://en.wikipedia.org/wiki/ISO_8601) date format is used? Would that be a better recommendation? If not, would the authors care to expand their argument for that as dates are used frequently in filenames?

 On page 9, (6) Naming symbols, (a) consider confining to lowercase letters.

 It seems to be rather arbitrary to confine names to lowercase, why would it not work for all uppercase letters instead?

 Similarly, in (b) avoid non-ASCII characters. Shouldn’t we be more inclusive of other languages that are non-ascii, e.g., European characters, or double byte Japanese, Korean and Chinese characters?

 From a computer coding point of view, I intuitively understand the rationale for choosing ASCII but the article doesn’t seem to provide a valid argument for it. May I suggest the authors to use international standard for POSIX Portable Operating System Interface (IEEE 1003 ISO/IEC 9945) (ref: https://en.wikipedia.org/wiki/POSIX; https://www.ibm.com/docs/en/zos/2.2.0?topic=locales-posix-portable-file-name-character-set) instead. Choosing to use an international standard makes more sense instead of creating another separate standard specifically for bioimaging data. If the authors would like to keep their recommendations, I would like to see more justification for doing so.

 On (d) upper limit on the length of file and folder names. The authors proposed a working upper limit of 50 characters. Again, it seems to be arbitrary, why not 80 characters, i.e. one line length on the old CRT terminal? The browser limit is a good reason, but I would like to see a more robust argument that 50 characters length is a good compromise.

 The authors used an example of file path limit of 320 characters in the same paragraph, I believe it may cause confusion for the reader with filename length, which for most computer systems, is only 255 characters. (ref: https://en.wikipedia.org/wiki/Filename ). Since the authors also provide recommendation (3) on Folder depth and given example of path length problems on page 7 “Very long names of files/folders”, maybe the authors can discuss and recommend that together, under one section “filename length, path length and folder depth”. It may be easier for the reader to appreciate the choice that the authors make.

 On recommendation (8) Friendly file formats. Maybe “Widely used file formats” is more applicable? I would prefer “Openly accessible file formats”, i.e., formats that there are readable by open-source tools.  I guess widely used file formats would fit that description too and reflect more closely to what the authors want to convey. Proprietary software tools for accessing proprietary file formats may cause problems in the long run as companies often change hands, e.g., Olympus is now Evident, LaVision is now under Brunker. It is difficult to ensure that companies will keep supporting certain formats in their software tools in the future while funding bodies (in the UK) require archiving data for 10 to 20 years.

Is the topic of the opinion article discussed accurately in the context of the current literature?

Yes

Are arguments sufficiently supported by evidence from the published literature?

Partly

Are all factual statements correct and adequately supported by citations?

Yes

Are the conclusions drawn balanced and justified on the basis of the presented arguments?

Yes

Reviewer Expertise:

Bioimage informatics

I confirm that I have read this submission and believe that I have an appropriate level of expertise to confirm that it is of an acceptable scientific standard, however I have significant reservations, as outlined above.

# Response

**Published:** 2024-01-30

**Authors:** Gerard J Kleywegt

**Affiliations:** EMBL European Bioinformatics Institute, UK

**Response to Reviewer #2** **(in italics)**

 Specific comments:

 The article is timely as we are facing a deluge of bioimaging data with higher resolutions and automation. It is therefore an area that needs more discussions, sharing of good practices.

 I mostly agree with all the recommendations given, although I feel that the authors may need to make a good argument for some recommendations. Some choices seem arbitrary and I would like to see the rationale  behind them.

 After reading the paper, I am a bit confused by the article’s intended target audience.

 Is the article recommendation aimed at most of the biologists who archive their bioimaging data mainly for the purpose of peer review and references? Or is the article and recommendation aiming for those database curators and producers of bioimaging databases, e.g. IDR ( https://idr.openmicroscopy.org/) , SSBD ( https://ssbd.riken.jp/database/), GDC ( https://portal.gdc.cancer.gov/) , etc?

 On page 4 under the heading ‘Motivation’, “We believe that recommendations outlined here maybe of value to two principal groups of users: 1) data depositors, who need to design and prepare their data to improve its usability to the community”.

 Does it include most biologists? I believe that most biologists archive their data to provide a record of their studies. Are the data depositors in the article and its intended audience refer to bioimaging database curators/producers instead of bench biologists?

 I believe that the ten recommendations would be equally applied to most biologists even though their aim is to provide a record of their studies, the recommendations would help those database curators to organise their bioimage data in more meaningful ways.

 I would like to see that part to be make clearer of its intended audience.

*We accept the correction and have expanded the introductory paragraphs to outline specific audiences as well as clarified the type of user that ‘user’ refers to.*

 With regards to the recommendations, on page 8, ‘Naming’ (5) Meaningful names (b) “Consider avoiding ambiguous attributes such as dates and times.

 The argument that they have “subtle variations” is not obvious to me.  Is it because of variations of date formats used in different countries? Would it be solved if ISO 8601 ( https://en.wikipedia.org/wiki/ISO_8601) date format is used? Would that be a better recommendation? If not, would the authors care to expand their argument for that as dates are used frequently in filenames?

*We accept the correction and have edited the text to better reflect the intended meaning.*

 On page 9, (6) Naming symbols, (a) consider confining to lowercase letters.

 It seems to be rather arbitrary to confine names to lowercase, why would it not work for all uppercase letters instead?

*We accept the correction and include arguments why we think it is preferable for file and folder names to be defined using lowercase letters.*

 Similarly, in (b) avoid non-ASCII characters. Shouldn’t we be more inclusive of other languages that are non-ascii, e.g., European characters, or double byte Japanese, Korean and Chinese characters?

 From a computer coding point of view, I intuitively understand the rationale for choosing ASCII but the article doesn’t seem to provide a valid argument for it. May I suggest the authors to use international standard for POSIX Portable Operating System Interface (IEEE 1003 ISO/IEC 9945) (ref: https://en.wikipedia.org/wiki/POSIX; https://www.ibm.com/docs/en/zos/2.2.0?topic=locales-posix-portable-file-name-character-set) instead. Choosing to use an international standard makes more sense instead of creating another separate standard specifically for bioimaging data. If the authors would like to keep their recommendations, I would like to see more justification for doing so.

*We accept the correction and now refer to POSIX as the standard to adhere to as well as provide reasons to do so.*

 On (d) upper limit on the length of file and folder names. The authors proposed a working upper limit of 50 characters. Again, it seems to be arbitrary, why not 80 characters, i.e. one line length on the old CRT terminal? The browser limit is a good reason, but I would like to see a more robust argument that 50 characters length is a good compromise.

*The reviewer’s comment does raise a valid point. However, it is important to bear in mind that file and folder names add to one another and a length of 80 means that at a depth of three folders will admit paths of up to 240 characters. It is hard to precisely determine what would be reasonable: 20-30 characters may be too short for a lot of cases. One option would be to examine file lengths in current archives to determine the distribution of file name lengths but if the objective is to follow good rather than current practice this may not be sound.*

*The authors propose the above limits to start a conversation with the community on what would be a sensible value or range.*

 The authors used an example of file path limit of 320 characters in the same paragraph, I believe it may cause confusion for the reader with filename length, which for most computer systems, is only 255 characters. (ref: https://en.wikipedia.org/wiki/Filename ). Since the authors also provide recommendation (3) on Folder depth and given example of path length problems on page 7 “Very long names of files/folders”, maybe the authors can discuss and recommend that together, under one section “filename length, path length and folder depth”. It may be easier for the reader to appreciate the choice that the authors make.

*We accept the correction and have restructured the article as suggested.*

 On recommendation (8) Friendly file formats. Maybe “Widely used file formats” is more applicable? I would prefer “Openly accessible file formats”, i.e., formats that there are readable by open-source tools.  I guess widely used file formats would fit that description too and reflect more closely to what the authors want to convey. Proprietary software tools for accessing proprietary file formats may cause problems in the long run as companies often change hands, e.g., Olympus is now Evident, LaVision is now under Brunker. It is difficult to ensure that companies will keep supporting certain formats in their software tools in the future while funding bodies (in the UK) require archiving data for 10 to 20 years.

*We have revised the section title to simply ‘File formats’. We appreciate that there are file formats that are unavoidable but proprietary (e.g., from microscopes) but our emphasis is on the openness of the formats because this enables the prevalence of tools which can reliably read the data. We have updated 8(b) to reflect this point.*

# Reviewer report: Reviewer response for version 1

**DOI:** 10.5256/f1000research.142422.r217553

**Published:** 2023-11-01

**Authors:** Sjors Scheres

**Affiliations:** 1 Medical Research Council Laboratory of Molecular Biology, Cambridge, England, UK

This paper describes recommendations for organizing imaging data from the life sciences for archival purposes. Coming from the EBI, which is responsible for a large proportion of image archiving in the field, this advice is important and worth of dissemination to the wider scientific community. I am therefore, in principle, enthusiastic about its publication in F1000Research. However, I do think that the manuscript and the explicit recommendations can be improved, as the phrasing is often vague and some of the recommendations are ignored by the authors themselves. I would therefore recommend a careful re-think and re-write, especially of the 10 recommendations, for a revised version.

 Specific comments:

**Abstract:**

 p1: The first sentence does not make sense: is 'organised data' an elusive goal?

 Motivation:

 p3: Would you not consider non-scientists looking at these images?

 p3: "in the use of 'ways and means' of effecting the organisation"

 -> I have no clue what this means.

 p4: "To achieve this ... and so on"

 -> These vague statements need rephrasing (e.g. 'we define [..] to the *various* attributes'). Also, what is 'generally available equipment'?

 p5: it is not entirely clear to me from reading the paper what the bandbox program does. The paper states that it is based on the 10 recommendations that follow, but as explained below the re-organisation in Figure 4 still violates several recommendations... Perhaps some pseudo-code may be useful? Also, wouldn't it make more sense to first describe the recommendations and then introduce this program?

**Recommendations:**

 Except for recommendation (7), all recommendations start with the word 'consider'. Given these are recommendations, that is superfluous. It may be clearer to use an imperative to directly state the recommendation (like done in 7).

 p6: How is a "raw" TIFF file defined?

 (3a) "the fewer the better" means a depth of 1 is best. This is probably not what the authors intended.

 (3b) Having a subfolder called 'tiff' is often a good idea, e.g. when there is also a file with metadata describing those tiff images (which is typically the case). In fact, the recommended Figure 4 has a 'raw' folder, which has exactly the same meaning, thus contradicting this recommendation. 

 (5a) What are "any references that are tied to the instrument" and why should they be excluded? If these are references to the microscope used, they may be relevant to the user?

 (5b) Why would dates in filenames be ambiguous and should they be avoided? Many data acquisition softwares write files with date and times in their names. Renaming these would, as the authors themselves point out, indeed be complicated and possibly lead to errors.

 (7a) I have no clue what this means: "similar folders at different depths have the same names"

 (7b) What are "personal identifiers"?

 (7c) The name 'data' is actually used in the line below and in the recommended Figure 4. Also, I don't see why 'images' won't be an excellent name for a folder that contains images?

 (7e) This recommendation may not be limited to slices of 3D data, which seems an arbitrarily narrow example for such broad recommendations. I personally thought of zer-padding images when I first read this (apparently not careful enough!). Perhaps using a term like "leading zeros" may be less ambiguous? Albeit perhaps useful to some of the readers, this is the only recommendation that has an explicit explanation of how to do this on two specific computer systems. Wouldn't this be something that could only be implemented in the bandbox program, so it could be used on any computer?

 (8) What are "friendly file formats?". Also, the term "widely used file formats" is not well defined.

 (9a) The example in Figure 4 does not have a README file...

 (9b) "This can be achieved ... data presents" -> This sounds superfluous and condescending.

 p11: The proposed path "data/brief_description/treatment3..." violates at least recommendations 3 (unused subfolder 'brief_description') and 7 (use of word 'data')

Is the topic of the opinion article discussed accurately in the context of the current literature?

Yes

Are arguments sufficiently supported by evidence from the published literature?

Yes

Are all factual statements correct and adequately supported by citations?

Partly

Are the conclusions drawn balanced and justified on the basis of the presented arguments?

Partly

Reviewer Expertise:

Structural biologist; software developer

I confirm that I have read this submission and believe that I have an appropriate level of expertise to confirm that it is of an acceptable scientific standard, however I have significant reservations, as outlined above.

# Response

**Published:** 2024-01-30

**Authors:** Gerard J Kleywegt

**Affiliations:** EMBL European Bioinformatics Institute, UK

**Response to Reviewer #1 (in italics)**

 Specific comments:

**Abstract:**

 p1: The first sentence does not make sense: is 'organised data' an elusive goal?

*Organised data is not in itself an elusive goal. However, when the volume and variety of data increase by orders or magnitude then maintaining organisation and coherence in the data is difficult to achieve and by extension makes the data difficult to use. Therefore, organised data - in the context of large heterogeneous datasets - is an elusive goal.*

 Motivation:

 p3: Would you not consider non-scientists looking at these images?

*In the article we use the term ‘scientist’ for anyone who aims to use data for some end. The claim is not that only scientists look at data; rather, anyone (formal scientist or not) who uses the data is referred to as a scientist. The order of terminology is important.*

 p3: "in the use of 'ways and means' of effecting the organisation"

 -> I have no clue what this means.

 p4: "To achieve this ... and so on"

 -> These vague statements need rephrasing (e.g. 'we define [..] to the *various* attributes'). Also, what is 'generally available equipment'?

*We have rephrased vague statements in line with this remark.*

 p5: it is not entirely clear to me from reading the paper what the bandbox program does. The paper states that it is based on the 10 recommendations that follow, but as explained below the re-organisation in Figure 4 still violates several recommendations... Perhaps some pseudo-code may be useful? Also, wouldn't it make more sense to first describe the recommendations and then introduce this program?

*We have moved the description of what bandbox does to the Software Availability section.*

**Recommendations:**

 Except for recommendation (7), all recommendations start with the word 'consider'. Given these are recommendations, that is superfluous. It may be clearer to use an imperative to directly state the recommendation (like done in 7).

 p6: How is a "raw" TIFF file defined?

*We have replaced this with the phrase ‘uncompressed TIFF files’.*

 (3a) "the fewer the better" means a depth of 1 is best. This is probably not what the authors intended.

*We accept the correction and have clarified the argument based on the ISA (investigation, study, assay) framework.*

 (3b) Having a subfolder called 'tiff' is often a good idea, e.g. when there is also a file with metadata describing those tiff images (which is typically the case). In fact, the recommended Figure 4 has a 'raw' folder, which has exactly the same meaning, thus contradicting this recommendation.

*We accept the correction and have revised the text for clarity that we are referring to intermediate folders where none are required.*

 (5a) What are "any references that are tied to the instrument" and why should they be excluded? If these are references to the microscope used, they may be relevant to the user?

*We accept the correction and have revised the phrase.*

 (5b) Why would dates in filenames be ambiguous and should they be avoided? Many data acquisition softwares write files with date and times in their names. Renaming these would, as the authors themselves point out, indeed be complicated and possibly lead to errors.

*The emphasis in the article is in having dates in folder names not file names. In (1b) we mention dates in file names as a possibility. Nevertheless, we do caution that date-time data on file names can also include subtle variations such as seconds so that numerous related files become non-trivial to work with due to these variations.*

 (7a) I have no clue what this means: "similar folders at different depths have the same names"

*We have revised the recommendation and included an example with reference to Figure 3.*

 (7b) What are "personal identifiers"?

*We have included a parenthetical remark with examples to illustrate what personal identifiers are.*

 (7c) The name 'data' is actually used in the line below and in the recommended Figure 4. Also, I don't see why 'images' won't be an excellent name for a folder that contains images?

*These examples are purely for illustration purposes but are inspired by the actual structure used in EMPIAR in which the ‘data’ directory sits beside an XML file e.g. https://ftp.ebi.ac.uk/empiar/world_availability/10002/, which we have omitted here. They were generated from the examples provided in the git repository.*

*We believe it is better to have descriptive folder names as opposed to generic names, which provide no meaningful information. The name ‘images’ does not convey any meaningful information. Better would be something like ‘tomograms’ or ‘particles’. Nevertheless, this is configurable in* *bandbox using the* *bandbox/obvious_files option in the configuration file.*

 (7e) This recommendation may not be limited to slices of 3D data, which seems an arbitrarily narrow example for such broad recommendations. I personally thought of zer-padding images when I first read this (apparently not careful enough!). Perhaps using a term like "leading zeros'' may be less ambiguous? Albeit perhaps useful to some of the readers, this is the only recommendation that has an explicit explanation of how to do this on two specific computer systems. Wouldn't this be something that could only be implemented in the bandbox program, so it could be used on any computer?

*We accept the correction and have rewritten the recommendation to clarify the context. We agree that implementing this in* *bandbox would enable a cross-platform solution and will plan this for a future release.*

 (8) What are "friendly file formats?". Also, the term "widely used file formats" is not well defined.

*This has been revised to simply ‘File formats’.*

 (9a) The example in Figure 4 does not have a README file…

*A README file has been added in the updated figure.*

 (9b) "This can be achieved ... data presents" -> This sounds superfluous and condescending.

*This sentence has been deleted in the article.*

 p11: The proposed path "data/brief_description/treatment3..." violates at least recommendations 3 (unused subfolder 'brief_description') and 7 (use of word 'data')

*As mentioned above, the example used here is purely illustrative and omits other content which would otherwise not violate this recommendation.*
